# Recent Developments on Five-Component Reactions

**DOI:** 10.3390/molecules26071986

**Published:** 2021-04-01

**Authors:** Xiaoming Ma, Sanjun Zhi, Wei Zhang

**Affiliations:** 1 School of Pharmacy, Changzhou University, 1 Gehu Road, Changzhou 213164, China; mxm.wuxi@cczu.edu.cn; 2 Jiangsu Key Laboratory for the Chemistry of Low-Dimensional Materials, Huaiyin Normal University, Huai’an 223300, China; jchyzhi2003@126.com; 3 Department of Chemistry, University of Massachusetts Boston, 100 Morrissey Boulevard, Boston, MA 02125, USA

**Keywords:** multicomponent reaction, five-component, pseudo, one-pot, cascade, consecutive, green synthesis, heterocycle, pot, atom, step economy

## Abstract

Multicomponent reactions (MCRs) have inherent advantages in pot, atom, and step economy (PASE). This important green synthetic approach has gained increasing attention due to high efficiency, minimal waste, saving resources, and straightforward procedures. Presented in this review article are the recent development on 5-compoment reactions (5CRs) of the following six types: (I) five different molecules A + B + C + D + E; pseudo-5CRs including (II) 2A + B + C + D, (III) 2A + 2B + C, (IV) 3A + B + C, (V) 3A + 2B, and (VI) 4A + B. 5CRs with more than five-reaction centers are also included.

## 1. Introduction

Reactions with a single operational step involving three or more components are called multicomponent reactions (MCRs) [1,2,3,4]. They have inherent advantages of pot, atom, and step economy (PASE) in the formation of multiple bonds of complex molecules [5,6]. MCRs integrate most of reactants to the product structures for mass efficiency, bypass the step of intermediate purification to reduce the amount of waste, and perform one-step reaction to simplify procedures and save resources. MCRs and associated one-pot synthesis and cascade reactions are active topics in the development of new methodologies in organic synthesis and catalysis [7,8].

**Figure 1:**
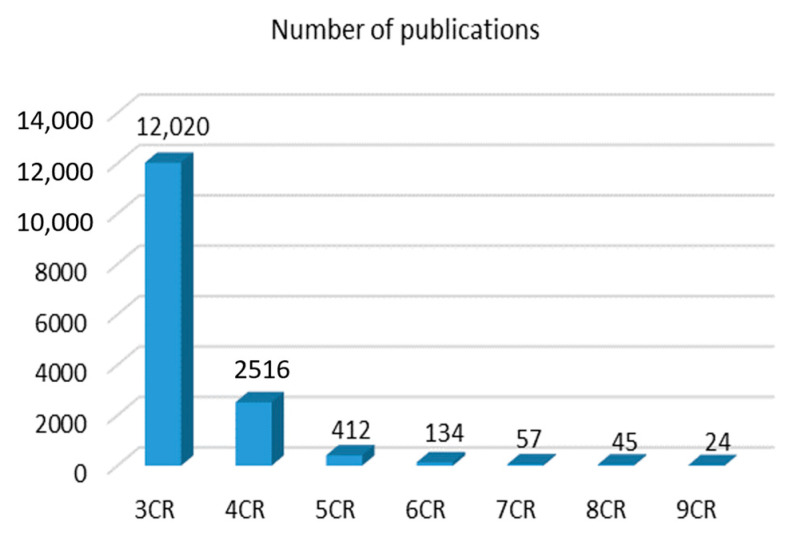
Publications of different MCRs.

There are couple dozen MCRs named after people, such as the well-known Ugi, Biginelli, Petasis, Hantzsch, Passerini, Huisgen, and Groebke-Blackburn-Bienaymé (GBB) reactions [1,2,3,4]. All these reactions are three- or four-component transformations. MCRs involving five or more components are called high-order MCRs, which are more efficient than regular MCRs in assembling complex structures [9]. However, the number of high-order MCRs is limited, because the increased number of competitive reactions for side-products make it harder to incorporate all the components in an orderly manner to form desirable products. Shown in Figure 1 is the distribution of MCRs from three up to nine components [10]. It shows a deep drop in paper numbers with the increase of reaction components.

**Figure 2:**
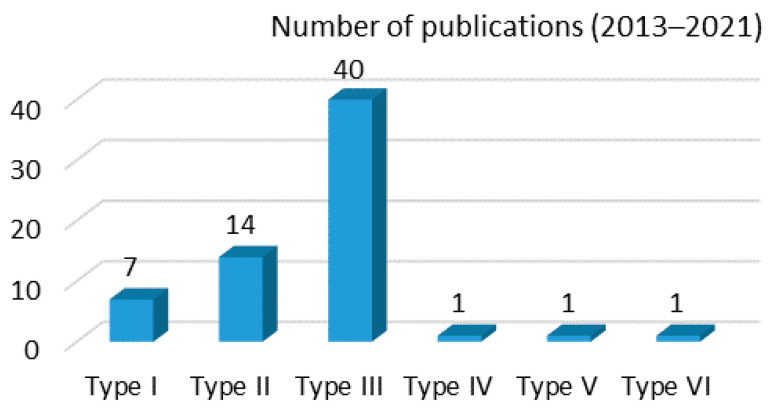
Papers of different 5MCRs covered in this work. Type I—A + B + C + D + E; Type II—2A + B + C + D; Type III—2A + 2B + C; Type IV—3A + B + C; Type V—3A + 2B; Type VI—4A + B.

There are numerous monographs and review articles on 3CRs and 4CRs [1,2,3,4], but only two reviews related to 5CRs in 2013 and 2020 [9,11]. Covered in this article are 5CRs mainly published after 2013. The 5CRs are classified into the following six types: (I) 5CR of five different components A + B + C + D + E; pseudo-5CRs of (II) 2A + B + C + D, (III) 2A + 2B + C, (IV) 3A + B + C, (V) 3A + 2B, and (VI) 4A + B. The number of these six kinds of 5CRs are quite different. Figure 2 shows the most popular 5CR is Type-III, followed by Type-II, and then Type-I. The number of the remaining types of MCRs is very limited.

If a component in the 5CRs has two reaction centers, then, the reaction is a 6-center 5-component reaction (6C5CR). It is important to note that MCRs only have a single operational step to charge all the components to the reaction vessel. If components are introduced in a stepwise manner at different stages of the reaction process, they should be called one-pot reactions instead of MCRs [7].

## 2. Type-I, 5CRs of A + B + C + D + E

**Scheme 1:**
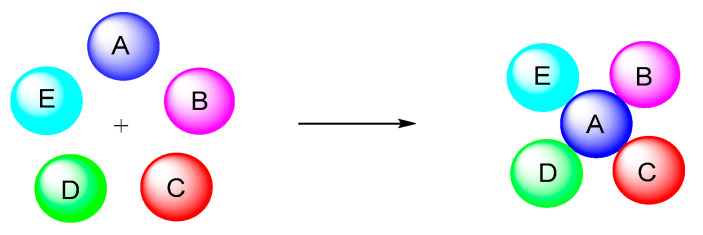
5CRs of A + B + C + D + E.

A schematic of a Type-I 5CRs involving five different molecules A + B + C + D + E is shown in Scheme 1. Because of complicated reaction mechanisms, the reported numbers of such reactions are limited. Since all components are different, the product structures could be unique and complex. A large number of analogs can be readily made by using different sets of starting materials.

**Scheme 2:**
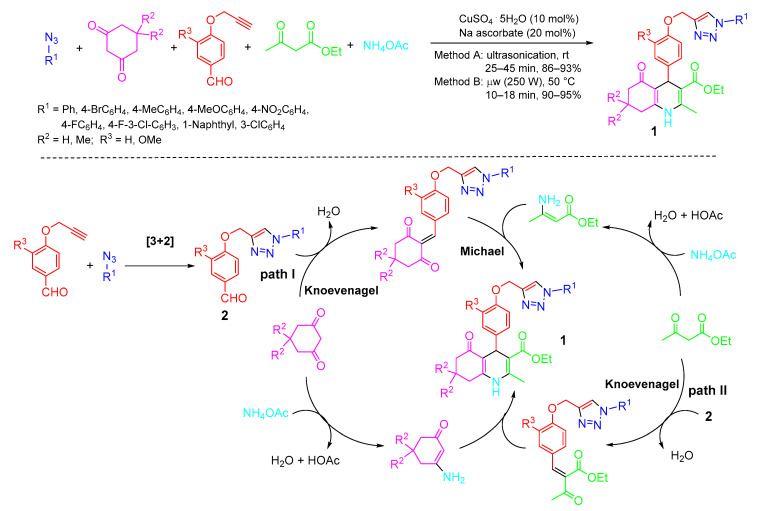
6C5CR for triazole-linked pentasubstituted 1,4-dihydropyridines.

Khurana and co-workers reported the synthesis of 1,2,3-triazole-linked 1,4-dihydropyridines **1** under ultrasonic or microwave irradiation using PEG-400 as a solvent (Scheme 2) [12]. The produced compounds were evaluated for antibacterial, antifungal, antioxidant activities, and also for photophysical properties. In the proposed reaction mechanism, the first step is a 1,3-dipolar cycloaddition of aryl azides and propargylated benzaldehydes to form 1,2,3-triazoles **2**. The next step (path I) is the Knoevenagel reaction of 1,2,3-triazoles **2** with 1,3-cyclohexanediones, followed by the Michael reaction of the enamine from ethyl acetoacetate and ammonium acetate to afford the products **1**. Another pathway (path II) is the Knoevenagel reaction of 1,2,3-triazoles **2** and ethyl acetoacetate, followed by Michael reaction of the enamine to afford products **1**. It is a 6C5CR, since propargylated benzaldehydes have 2-reaction centers.

**Scheme 3:**
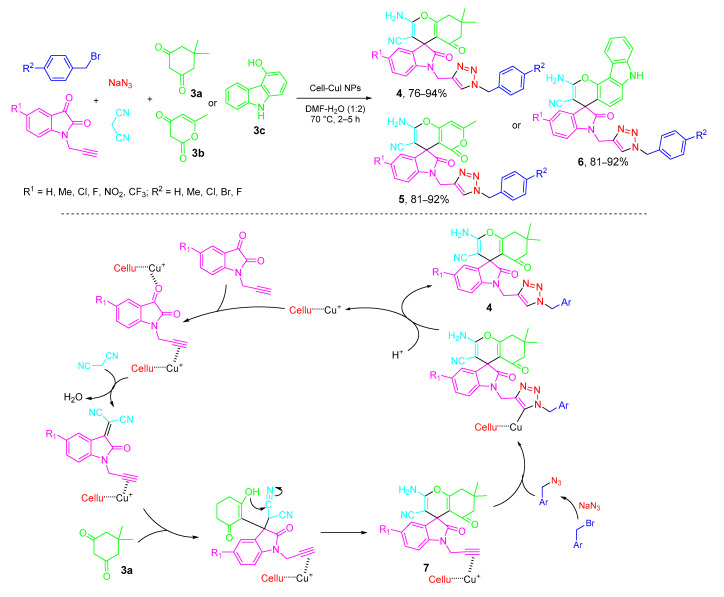
6C5CR for spirochromenocarbazole-tethered 1,2,3-triazoles.

Desai and co-workers introduced a reaction of benzyl halides, *N*-propargyl isatins, sodium azide, malononitrile, and 5,5-dimethyl-1,3-cyclohexanedione (**3a**), 6-methyl-2*H*-pyran-2,4(3*H*)-dione (**3b**) or 4-hydroxycarbazole (**3c**) for the synthesis of 1,2,3-triazole-tethered spirochromenocarbazoles **4**, **5** or **6** using cellulose-supported CuI nanoparticles (Cell-CuI NPs) as a reusable catalyst (Scheme 3) [13,14]. The first step is the condensation of *N*-propargyl isatins, malononitrile and **3a**, followed by cyclization to form spirochromenes **7**. The Huisgen 1,3-dipolar cycloaddition of **7** with benzylic azides affords 1,2,3-triazoles **4**. The Cell-CuI NPs catalyst can be recovered for reuse. It is a 6C5CR since *N*-propargyl isatins have 2-reaction centers. All the synthesized products have been screened against *Mycobacterium tuberculosis* H37Ra (ATCC 25177), *Mycobacterium bovis* BCG (ATCC 35743), as well as panel of cancer cell lines. Some products exhibited antimycobacterial, antitubercular, antibacterial, and anti-proliferative activities.

**Scheme 4:**
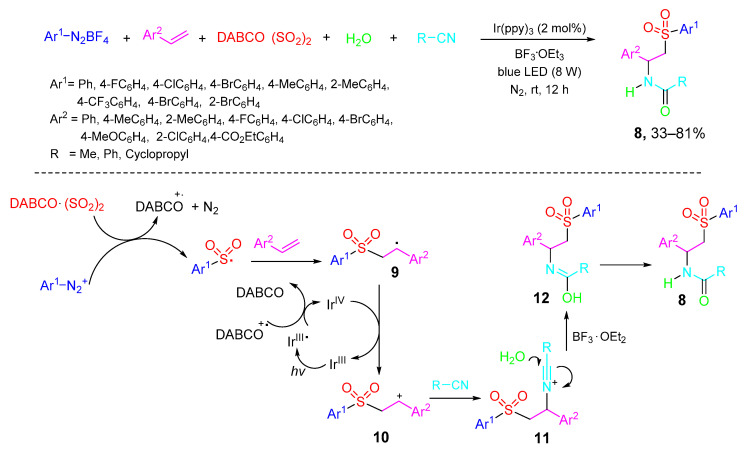
5CR for β-sulfonyl amides.

Wu and co-workers reported a photocatalytic reaction of aryldiazonium tetrafluoroborates, styrenes, sulfur dioxide, water, and nitriles for the synthesis of β-sulfonyl amides **8** at room temperature (Scheme 4) [15]. The vicinal aminosulfonylation of styrenes with the insertion of sulfur dioxide proceeded smoothly to give β-sulfonyl amides **8**. The aryl radical generated from the reaction of aryldiazonium tetrafluoroborate and DABCO·(SO_2_)_2_ is captured by SO_2_ to form arylsulfonyl radical which then attacks the terminal position of the styrenes to provide intermediate radicals **9**. The excited Ir-photocatalyst oxidizes the radicals to cations **10** through a single electron transfer (SET) mechanism. The nitriles as nucleophiles react with cations **10** to form **11** and then **12** in the presence of Lewis acid and H_2_O to afford products **8** after isomerization.

**Scheme 5:**
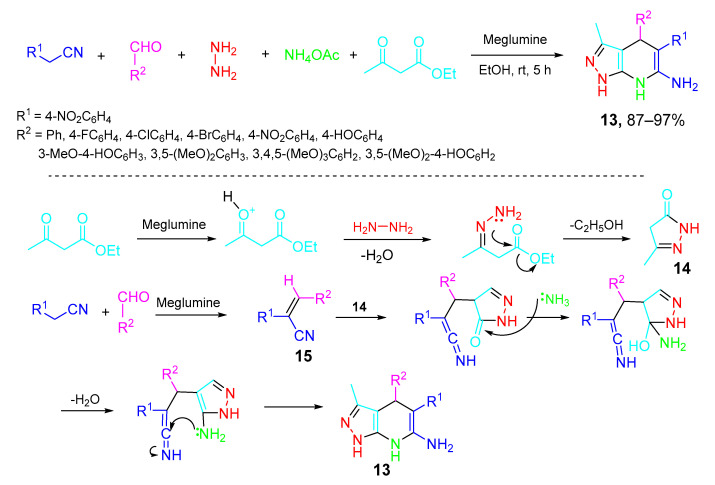
6C5CR for 4,7-dihydro-1*H*-pyrazolo[3,4-*b*]pyridin-6-amines.

Pasha and co-workers developed a reaction of substituted phenylacetonitriles, aryl aldehydes, hydrazine, ammonium acetate, and ethyl acetoacetate for the synthesis of 4,7-dihydro-1*H*-pyrazolo [3,4-*b*]pyridin-6-amines **13** under the catalysis of meglumine (Scheme 5) [16]. Meglumine has ammonium and alkoxy groups which can activate ethyl acetoacetate and phenylacetonitriles through hydrogen bonding and also donate electrons from the oxygen atom. As shown in the proposed mechanism, the protonated ethyl acetoacetate reacts with hydrazine to yield **14**. The aryl acetonitriles undergo a Knoevenagel condensation with aryl aldehydes to form α,β-unsaturated nitriles **15**. The Michael addition of **14** and **15** followed by the nucleophilic attack of NH_3_ and cyclization afford products **13**. It is a 6C5CR, since hydrazine is a 2-centered reactant.

**Scheme 6:**
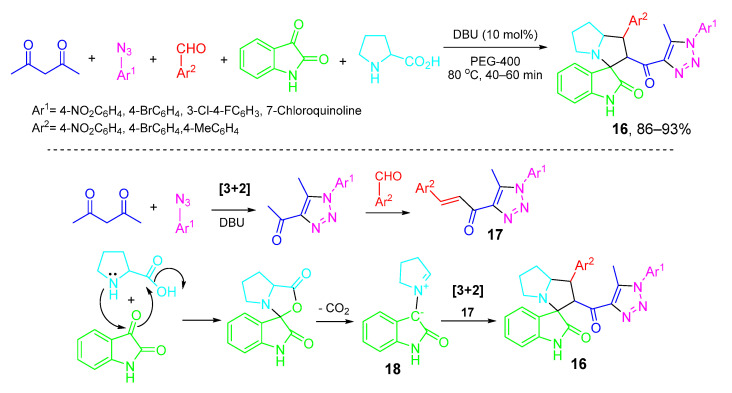
5CR for heterocyclic triazolyl spirocyclic oxindoles.

Khurana and co-workers reported a reaction of acetylacetone, aryl azides, aryl aldehydes, isatin, and L-proline for the synthesis of novel heterocyclic triazolyl spirooxindoles **16** (Scheme 6) using DBU as a catalyst and PEG-400 as a solvent [17]. In the reaction process, the triazoles generated from a [3+2] cycloaddition of acetylacetone and azides undergo aldol condensation with aryl aldehydes to give chalcone derivatives **17**. Condensation of isatin and L-proline, followed by decarboxylation give ylide **18**. The final step, a [3+2] cycloaddition of **17** and **18**, gives products **16**.

**Scheme 7:**
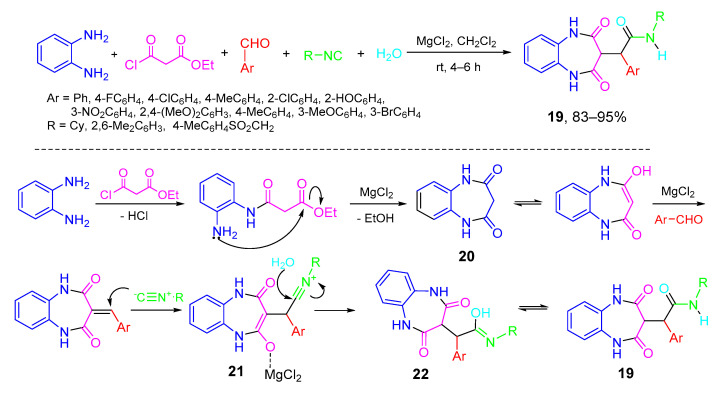
5CR for benzodiazepine derivatives.

Rahmati and co-workers introduced a reaction of *o*-benzenediamine, ethyl malonyl chloride, aromatic aldehydes, isocyanides, and water for the synthesis of benzodiazepines **19** using MgCl_2_ as a catalyst and CH_2_Cl_2_ as the solvent (Scheme 7) [18]. The formation of the amide through reaction of *o*-benzenediamine and ethyl malonyl chloride is followed by cyclization to afford seven-membered ring **20** under the catalysis by MgCl_2_. Then, Knoevenagel condensation of seven-membered ring and aldehyde leads to α,β-unsaturated molecules **21**, which undergo Michael-type addition with isocyanides to form **21** followed by hydrolysis which leads to compounds **22**. The final isomerization of compounds **22** gives diazepine amides **19**.

## 3. Type-II, Pseudo-5CRs of 2A + B + C + D

**Scheme 8:**
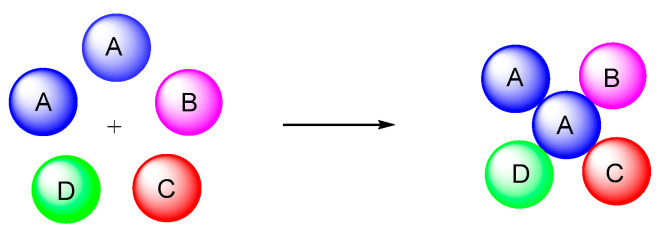
5CRs of 2A + B + C + D.

A schematic of a Type-II pseudo-5CRs involving 2A + B + C + D is shown in Scheme 8. Two molecules of component A are involved in the reaction process and are incorporated into the product.

**Scheme 9:**
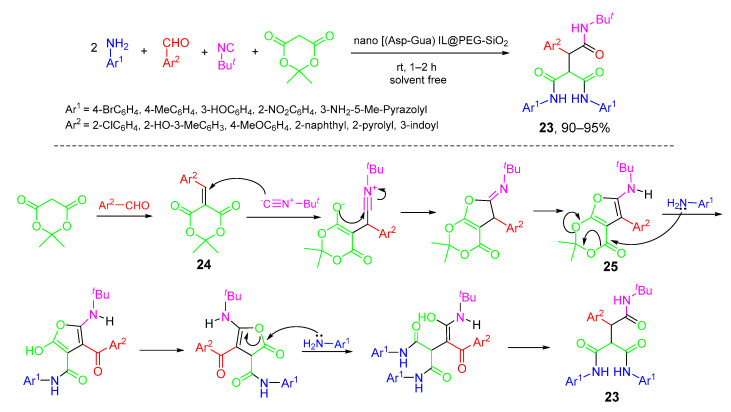
Pseudo-5CR for tricarboxamide derivatives.

Nikoofar and co-workers embedded aspartic acid-guanine ionic liquid on the hydroxylated nano silica face for making a novel bio-based core-shell organic–inorganic nano catalyst (Asp-Gua) IL@PEG-SiO_2_, and employed it for the synthesis of tricarboxamide derivatives **23** using two equiv of aromatic amines and one equiv each of aromatic aldehydes, *t*-butyl isocyanide, and Meldrum’s acid (Scheme 9) [19]. The reaction starts with the Knoevenagel condensation of Meldrum’s acid and aldehydes to form α,β-unsaturated compounds **24**. Michael-type addition of isocyanides to **24** followed by intermolecular nucleophilic attack lead to compounds **25**. Amidation of **25** with aromatic amines and sequential isomerization produce tricarboxamides **23**.

**Scheme 10:**
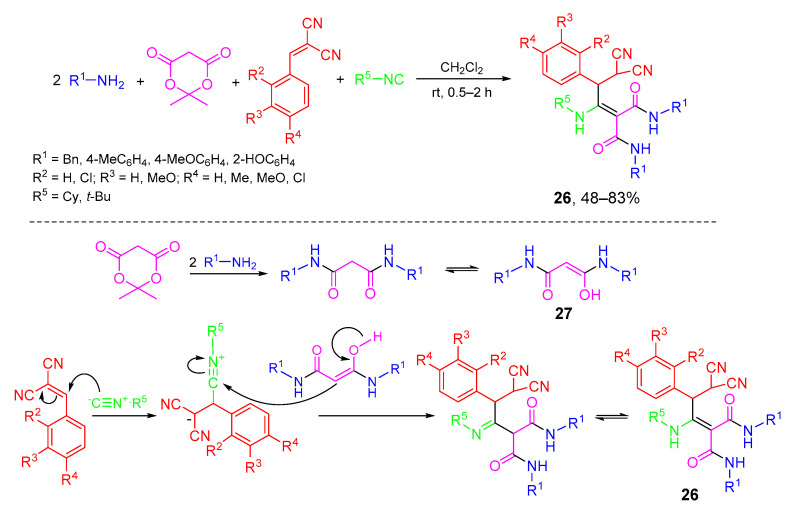
Pseudo-5CR for malonamides.

Rahmati and coworkers developed a method for the synthesis of malonamides **26** with two equiv of amine and one equiv each of Meldrum’s acid, arylidene malononitrile, and isocyanide in CH_2_Cl_2_ at ambient temperature (Scheme 10) [20]. The synthesis involves the nucleophilic attack of isocyanides to arylidene malononitriles followed by the nucleophilic attack of malonamides **27** produced from reaction between a Meldrum’s acid and two molecules of amine to give the final products after tautomerization.

**Scheme 11:**
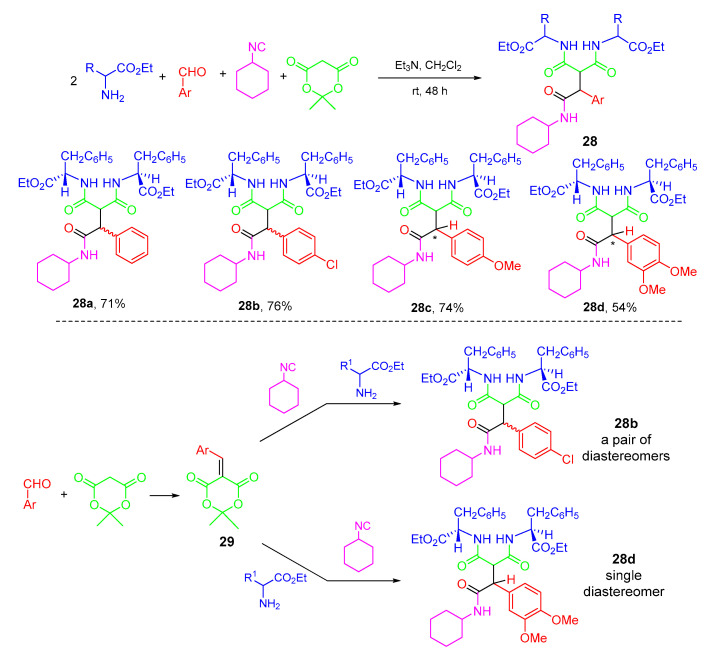
Pseudo-5CR for malonamide pseudopeptidic compounds.

Rahmati and co-workers reported the synthesis of malonamides pseudopeptidic compounds **28** via the reaction of two equiv of amino esters, one equiv each of aromatic aldehydes, isocyanide, and Meldrum’s acid (Scheme 11) [21]. Unsubstituted and electron-deficient aryl aldehydes produce a mixture of two diastereomers such as **28a** and **28b**, while the electron-rich aryl aldehydes produce only one isomer **28c** or **28d**. The results could be explained by the order of reactants participation in the reaction. In the reaction of unsubstituted and electron-poor aryl aldehydes, isocyanide reacts with the arylidene Meldrum’s acid **29** and then, with amino esters to give **28b** as a mixture of two diastereomers. In the reaction of electron-rich aryl aldehydes, amino esters react with arylidene Meldrum’s acid **29** before the isocyanide to give **28d** as a single diastereomer due to the chirality effect of the amino esters.

**Scheme 12:**
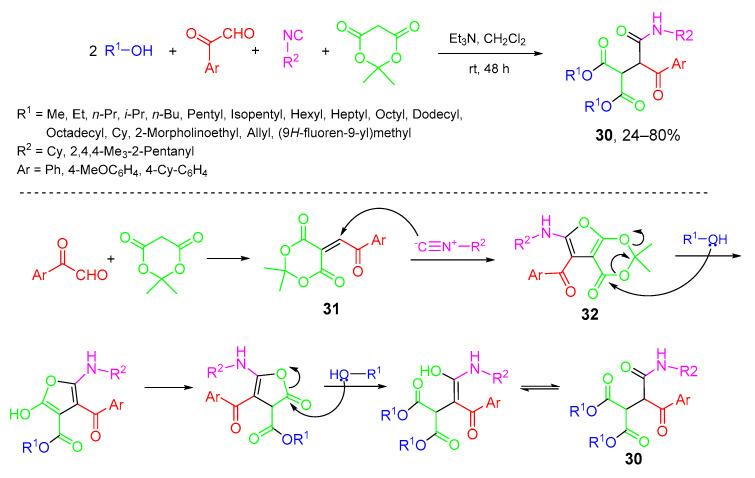
Pseudo-5CR for malonates.

Rahmati and Googol reported a synthesis of dialkyl 2-(1-(alkylamino)-1,3-dioxo-3-phenylpropan-2-yl)malonates **30** using two equiv of alcohols, one equiv each of aryl glyoxals, isocyanides and Meldrum’s acid (Scheme 12) [22]. The synthesis involves Knoevenagel condensation of 2-oxo-2-phenyl acetaldehydes and Meldrum’s acid to give **31**, followed by Michael-type addition of isocyanides and cyclization to form aminofurans **32.** Further reaction of **32** with two equiv of alcohols then afforded products **30** after tautomerization. The products could be used as low molecular weight supramolecular organogelators.

**Scheme 13:**
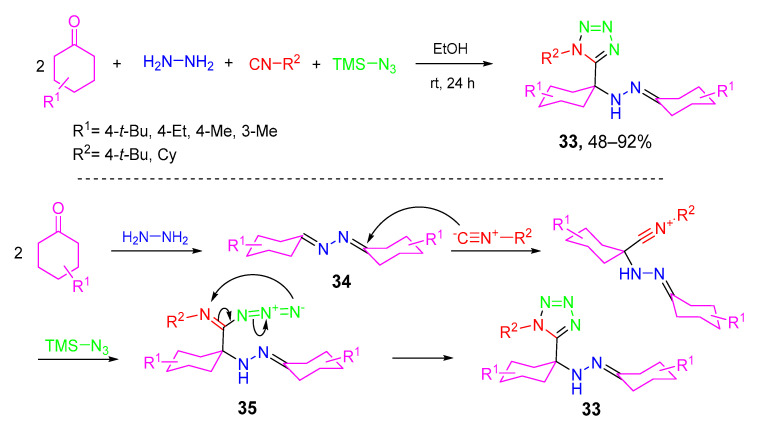
Pseudo-5CR for the synthesis of α-hydrazino tetrazoles.

Balalaie and co-workers developed a reaction of two equiv of cyclic ketones and one equiv each of hydrazine hydrate, trimethylsilyl azide, and isocyanides *α*-hydrazino tetrazoles for the synthesis of **33** (Scheme 13) [23]. The reaction process starts with the condensation of two molecules of the cyclic ketones with hydrazine hydrate to form dicycloalkydiimines **34**. Nucleophilic additions of isocyanides and then trimethylsilyl azide to **34** give intermediates **35** which undergo dipolar cyclization to provide products **33**.

**Scheme 14:**
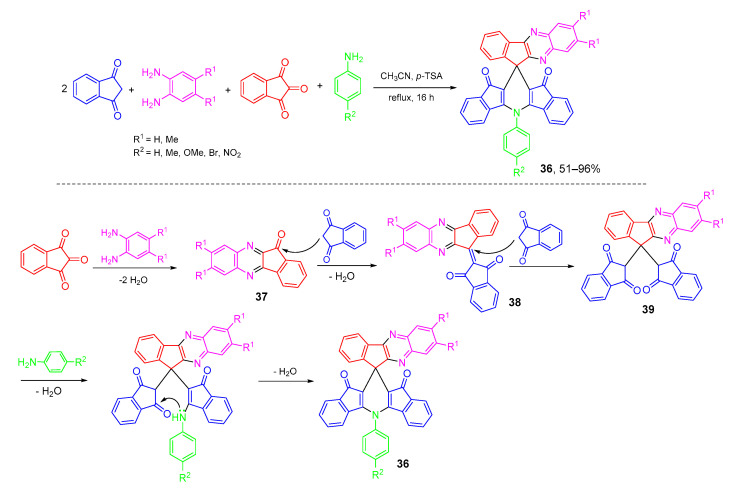
Pseudo-5CR for 5-phenyldihydrospiro(diindenopyridine-indenoquinoxaline) diones.

Ghahremanzadeh and co-workers reported a reaction of two equiv of 1*H*-indene-1,3(*2H*)-dione and one equiv each of *o*-benzenediamines, 1*H*-indene-1,2,3-trione and anilines under the catalysis of *p*-TSA to give 5-phenyldihydrospiro-(diindenopyridine-indenoquinoxaline) diones **36** (Scheme 14) [24]. The reaction starts with the iminization-aromatization reaction of 1*H*-indene-1,2,3-trione with *o*-benzenediamine to afford **37,** followed by Knoevenagel condensation with 1*H*-indene-1,3(2*H*)-dione to form **38**, and then Michael-type addition with a second 1*H*-indene-1,3(2*H*)-dione to produce intermediates **39**. Finally, the reaction of **39** with anilines followed by cyclization and tautomerization afford products **36**.

**Scheme 15:**
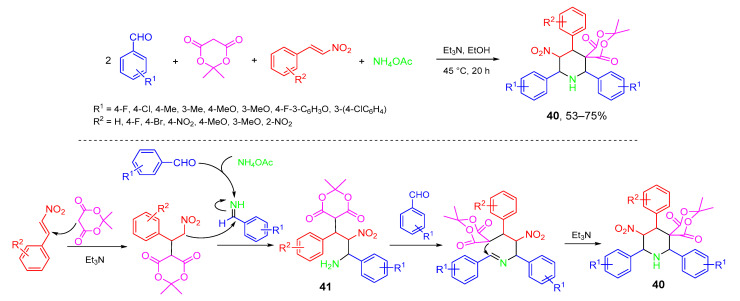
Pseudo-5CR for the synthesis of highly functionalized piperidines.

Wang and co-workers developed a reaction for the synthesis of highly functionalized piperidines **40** using two equiv of aromatic aldehydes, and one equiv each of Meldrum’s acid, substituted β-nitrostyrenes and ammonium acetate under basic conditions (Scheme 15) [25]. First, the Michael addition of Meldrum’s acid to substituted nitrostyrenes followed by nitro-Mannich nucleophilic addition on intermediate arylimine gives amines **41**. Second aromatic aldehydes react with amines **41** followed by intramolecular nitro-Mannich nucleophilic addition to give cyclic amines **40**.

**Scheme 16:**
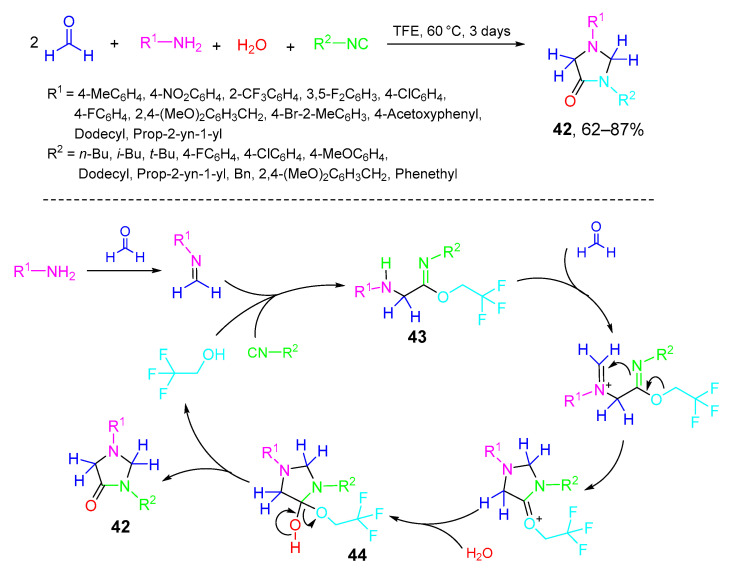
Pseudo-5CR for 4-imidazolidinones.

Ramírez and co-workers developed a reaction using two equiv of formaldehyde and one equiv each of primary amine, water and isocyanide for the synthesis of *N*,*N*′-substituted 4-imidazolidinones **42** (Scheme 16) [26]. Trifluoroethanol (TFE) was used as both a solvent and a reagent. Imines generated in situ from formaldehyde and amines react with isocyanides and TFE to give amines **43** which then react with second formaldehyde followed by an intramolecular nucleophilic attack and addition of water to form hemiorthoamides **44**. Releasing of TFE from **44** gives 4-imidazolidinone **42**.

**Scheme 17:**
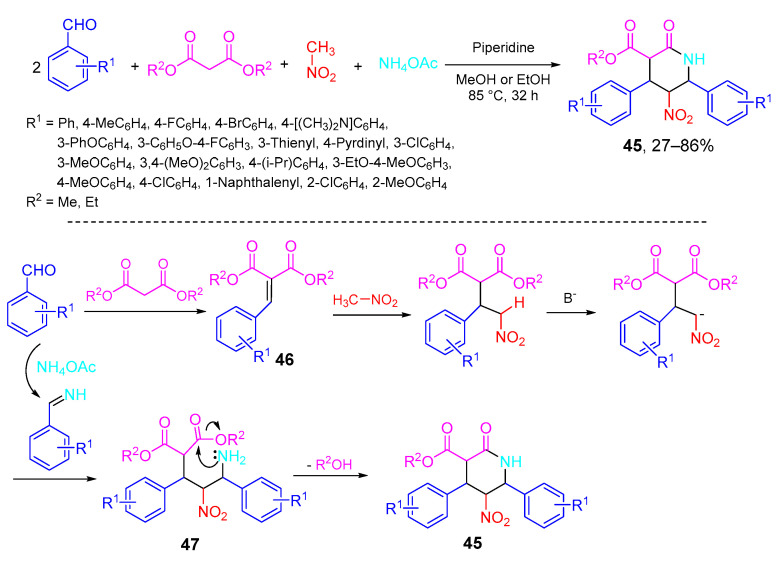
Pseudo-5CR for polysubstituted 2-piperidinones.

Wang and co-workers reported a method for the diastereoselective synthesis of polysubstituted 2-piperidinones **45** using two equiv of aromatic aldehydes and one equiv each of dialkyl malonates, nitromethane and ammonium acetate (Scheme 17) [27]. The reaction involves Michael addition of the nitromethane to the arylidene malonates **46**, followed by nucleophilic addition to arylimines generated from the reaction of aromatic aldehydes and ammonium acetate to form intermediates **47** which undergo lactamization to give *trans* isomer cyclic piperidinones **45** (racemic) after eliminating the alcohol.

**Scheme 18:**
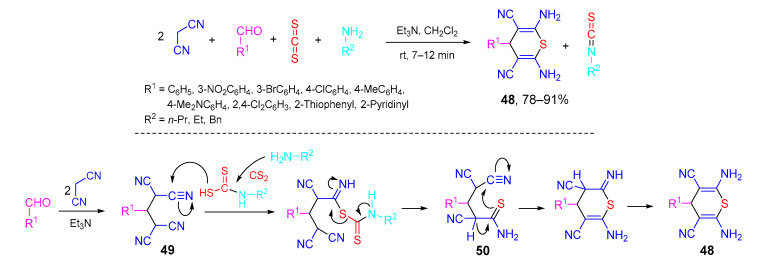
Pseudo-5CR for substituted 4*H*-thiopyrans.

Bodaghifard and co-workers reported a method for the synthesis of substituted 4*H*-thiopyrans **48** involving two equiv of malononitrile and one equiv each of aldehydes, carbon disulfide and primary amines under the catalysis of Et_3_N (Scheme 18) [28]. The reaction involves the Knoevenagel reaction of aldehydes and malononitrile followed by Michael addition to form **49**. Nucleophilic attack on **49** by aminodithioic acids generated from the reaction of amines and carbon disulfide followed by H-transfer and carbon–sulfur bond cleavage gives isothiocyanates **50**. H-shift of **50** and cyclization followed by another H-shift give substituted 4*H*-thiopyrans **48**. This reaction involves five components, but no fragment from the primary amines remains in the products, so primary amines are used as a reagent, not a reactant.

**Scheme 19:**
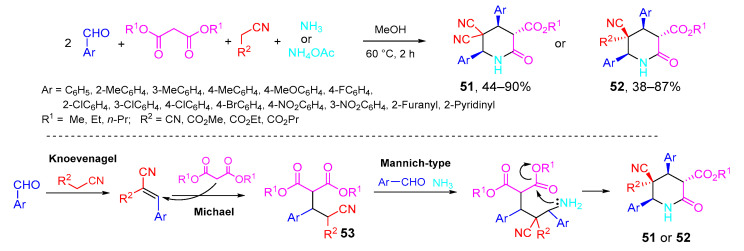
Pseudo-5CR for 2-piperidinone derivatives.

Vereshchagin and co-workers developed a reaction using two equiv of aryl aldehydes and one equiv each of dialkylmalonates, malononitrile or alkyl cyanoacetate, and ammonium acetate or ammonia for the synthesis of 2-piperidinone derivatives **51** or **52** (Scheme 19) [29]. The reaction involves Knoevenagel condensation of aryl aldehydes with malononitrile or alkyl cyanoacetate followed by Michael addition of dialkylmalonates to afford intermediates **53**. Then, Mannich-type condensation of **53**, aryl aldehydes and ammonium acetate followed by lactamization afford the corresponding 2-piperidinone derivatives **51** or **52**.

**Scheme 20:**
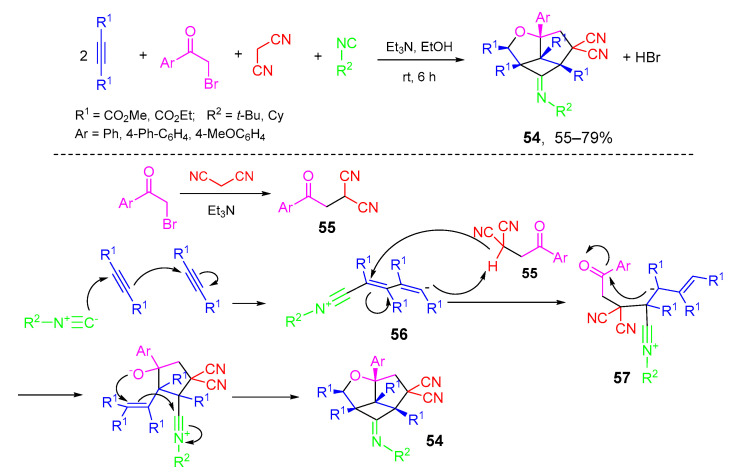
Pseudo-5CR for 3-oxacyclobuta[*cd*]pentalenes.

The Adib lab developed a method for the synthesis of 3-oxacyclobuta[*cd*]pentalenes **54** using two equiv of dialkyl acetylenedicarboxylates and one equiv each of phenacyl bromides, malononitrile and isocyanides at ambient temperature in absolute ethanol (Scheme 20) [30]. The phenacyl bromides undergo nucleophilic substitution with malononitrile in the presence of Et_3_N to form malononitriles **55** for the reaction with zwitterionic intermediates **56** which are generated in situ from the reaction of the isocyanides and the dialkyl acetylenedicarboxylates. The resulting adducts **57** undergo cyclization followed by conjugate addition to afford the products **54**.

**Scheme 21:**
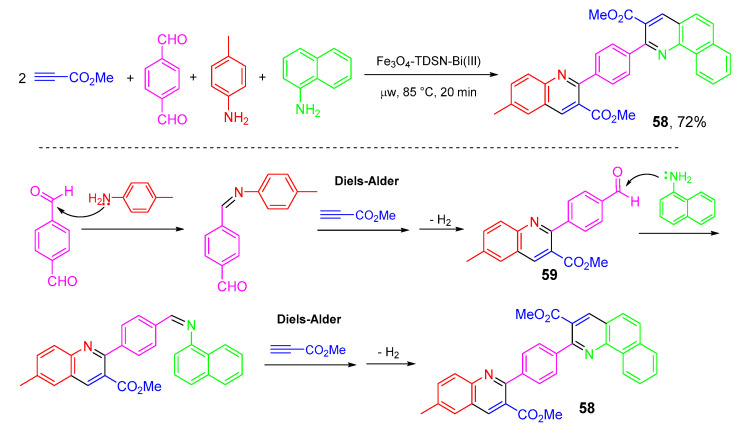
6C5CR for a biquinoline.

Mohammadpoor-Baltork and co-workers reported a method for the synthesis of biquinoline **58** employing two equiv of methyl propiolate and one equiv each of terephthaldialdehyde, naphthalen-1-amine, and *p*-toluidine using reusable Fe_3_O_4_-TDSN-Bi(III) catalyst (Bi(III) immobilized on triazine dendrimer-stabilized magnetic nanoparticles) under microwave heating and solvent-free conditions (Scheme 21) [31]. The synthesis may involve the condensation of terephthaldialdehyde and *p*-toluidine followed by Diels–Alder reaction and aromatization to form **59**. The reaction of **59** and naphthalen-1-amine followed by another Diels–Alder reaction affords biquinoline **58** after aromatization. It is a 6C5CR, since terephthaldialdehyde is a 2-centered reactant.

**Scheme 22:**
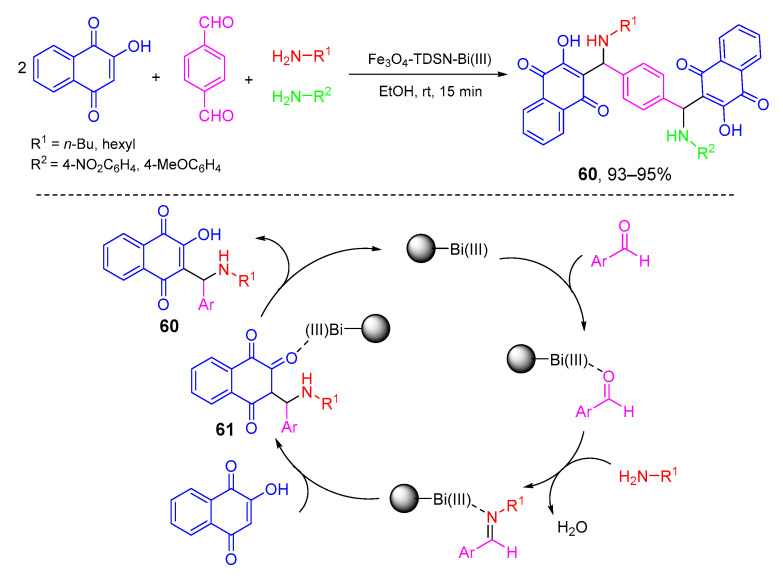
6C5CR for bisaminonaphthoquinones.

Mohammadpoor-Baltork and co-workers developed a method for the synthesis of aminonaphthoquinones **60** by reacting two equiv of 2-hydroxynaphthalene-1,4-dione with one equiv each of terephthaldialdehyde, alkylamines and arylamines using Fe_3_O_4_-TDSN-Bi(III) as a reusable catalyst (Scheme 22) [32]. The reaction process involves activation of terephthaldialdehydes with Fe_3_O_4_-DSN-Bi(III) followed by condensation with amines and addition of 2-hydroxynaphthalene-1,4-dione to give products **61** after tautomerization and releasing of catalyst Fe_3_O_4_-DSN-Bi(III). It is a 6C5CR, since terephthaldialdehyde is a 2-centered reactant.

## 4. Type-III, Pseudo-5CRs of 2A + 2B + C

**Scheme 23:**
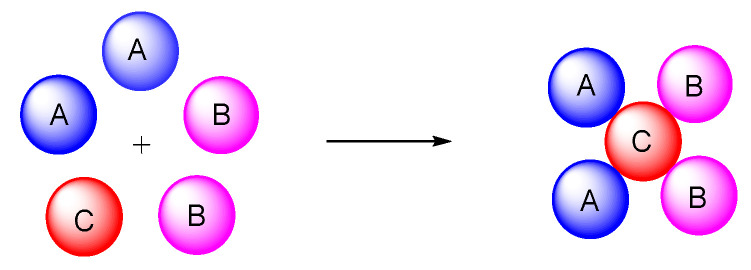
5CRs of 2A + 2B + C.

A schematic of a Type-III pseudo-5CRs involving 2A + 2B + C is shown in Scheme 23. Two molecules each of components A and B are involved in the reaction with one equiv of compound C. In many cases, component C is a two-centered reactant which makes the reaction classify as a 6C5CR. As shown in Figure 2, Type-III reactions are the most popular 5CRs. Product structures could be symmetrical, especially from the 6C5CRs.

**Scheme 24:**
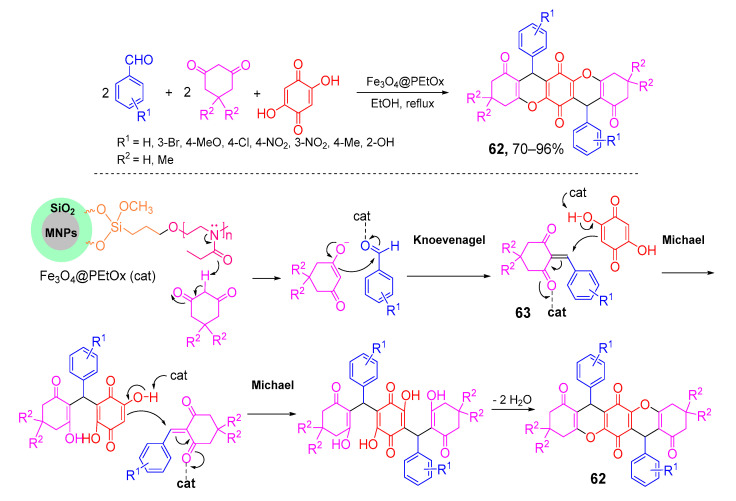
Pseudo-5CR for tetrahydrochromeno[2,3-*b*]xanthene tetraones.

The Moradi group employed immobilized poly(2-ethyl-2-oxazoline) (PEtOx) nanoparticles (Fe_3_O_4_@SiO_2_/PEtOx) for the synthesis of tetrahydrochromeno[2,3-*b*]xanthene tetraones **62** using two equiv each of arylaldehydes and 1,3-cyclohexanediones and one equiv of 2,5-dihydroxy-1,4-benzoquinone (Scheme 24) [33]. The amide bands in PEtOx catalyze the Knoevenagel condensation of 1,3-cyclohexanediones and benzaldehydes to afford intermediates **63** which then undergo double Michael additions followed by cyclization and dehydration to give products **62**. The magnetic nanocatalyst could be easily separated by an external magnet and reused for five runs without significant loss of activity.

Pyrazoles, such as bis(1*H*-pyrazol-5-ols), are important fragments in drug molecules. A common method for the synthesis of 4,4′-(arylmethylene)bis(1*H*-pyrazol-5-ol) derivatives is to conduct a pseudo-5CR of two equiv each of phenylhydrazines and ethyl acetoacetate with one equiv of aromatic aldehydes through a cycloaddition-Knoevenagel-Michael reaction sequence. In recent years, the synthesis of bis(1*H*-pyrazol-5-ols) has been accomplished using different catalysts, such as nanocatalyst Pd(0)-guanidine@MCM-41 [34], guanidine hydrochloride [35], *α*-Casein [36], ZnAl_2_O_4_ nanoparticle [37], La(OTf)_2_-grafted-GO (graphene oxide) [38], amino acid-based ionic liquids (AAILs) [39], and CuCr_2_O_4_ nanoparticle [40]. Catalyst-free conditions have also been reported [41].

**Scheme 25:**
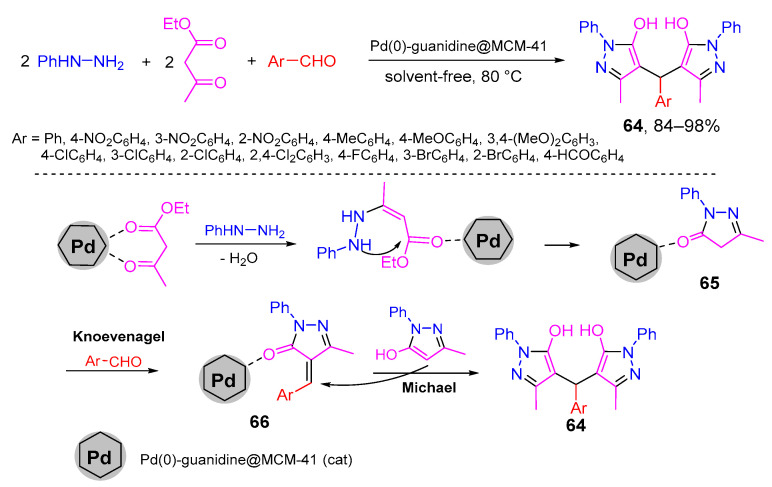
Pseudo-5CR for 4,4′-(arylmethylene)bis(1*H*-pyrazol-5-ol) derivatives.

Shown in Scheme 25 is an example for the synthesis of 4,4′-(arylmethylene)-bis(1*H*-pyrazol-5-ols) **64** through a pseudo-5CR. The reaction reported by the Filian group employed two equiv each of phenylhydrazines and ethyl acetoacetate and one equiv of aromatic aldehydes in the presence of Pd(0)-guanidine@MCM-41 as a nanocatalyst [34]. The carbonyl groups of ethyl acetoacetate are activated by the Pd-nanocatalyst for the reaction with phenyl hydrazine to afford pyrazolone **65** which then undergoes Knoevenagel-type reaction to give intermediate **66** for Michael addition with pyrazolone tautomer to afford bis(1*H*-pyrazol-5-ols) **64**.

**Scheme 26:**
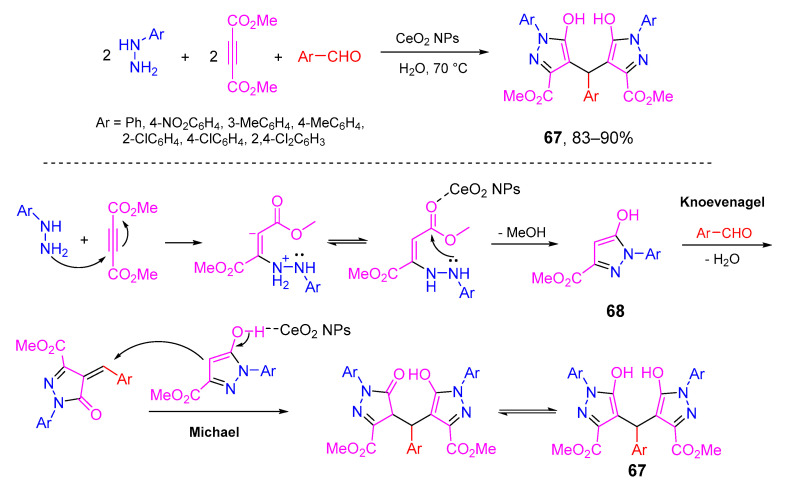
Pseudo-5CR for the synthesis of bis(pyrazol-5-ol) derivatives.

Safaei-Ghomi and coworkers developed a method for the synthesis of bis(pyrazol-5-ol) derivatives **67** using two equiv each of arylhydrazine, acetylenedicarboxylates and one equiv of aromatic aldehydes under the catalysis of CeO_2_ nanoparticles (Scheme 26) [42]. Nucleophilic reaction of arylhydrazine with acetylenedicarboxylates followed by cyclization form pyrazolone intermediate **68.** Knoevenagel condensation of **68** and aromatic aldehydes followed by Michael addition with **68** afford bis(pyrazol-5-ol) derivatives **67**. The Xu group conducted a similar reaction using Dabco-base ionic liquid as a catalyst [43].

**Scheme 27:**
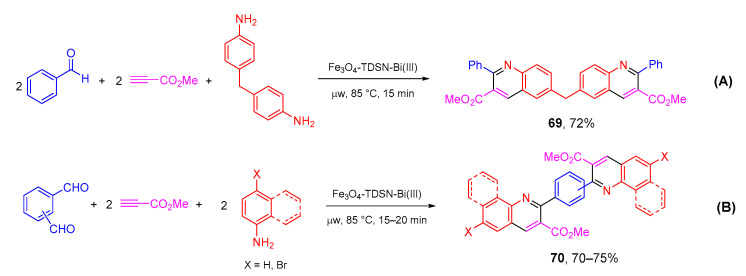
6C5CR for symmetric bisquinolines. (**A**) Diamines involved reaction. (**B**) Dialdehydes involved reaction.

Mohammadpoor-Baltork and co-workers extended the 2A + B + C + D type pseudo-5CR shown in Scheme 21 to a 2A + 2B + C type by using diamines (Scheme 27A) or dialdehydes (Scheme 27B) as two-centered reactants in the synthesis of symmetric bisquinolines **69** and **70 [31]**. It only took 15–20 min for accomplishing the reaction under microwave irradiation condition.

**Scheme 28:**
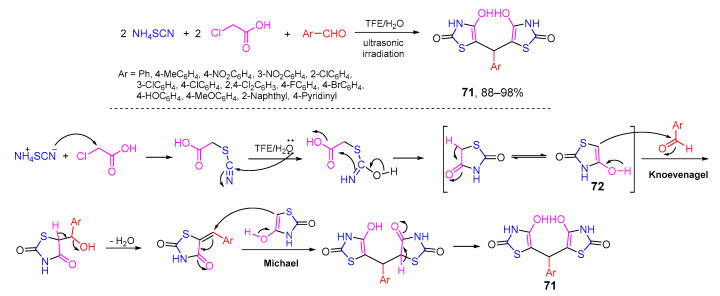
Pseudo-5CR for 5,5′-(arylmethylene)bis(4-hydroxythiazole-2(3*H*)-one) derivatives.

Heravi and coworkers developed a method for the synthesis of 5,5′-(arylmethylene)-bis(4-hydroxythiazole-2(3*H*)-one) derivatives **71** by reacting one equiv of aryl aldehydes and two equiv each of monochloroacetic acid and ammonium thiocyanate in TFE/water (1:1) under ultrasound irradiation at room temperature (Scheme 28) [44]. In this reaction, the condensation between monochloroacetic acid and ammonium thiocyanate followed by hydrolysis and cyclization affords thiazolone **72**. Knoevenagel condensation of **72** with aromatic aldehydes followed by Michael addition and tautomerization then affords final products **71**.

**Scheme 29:**
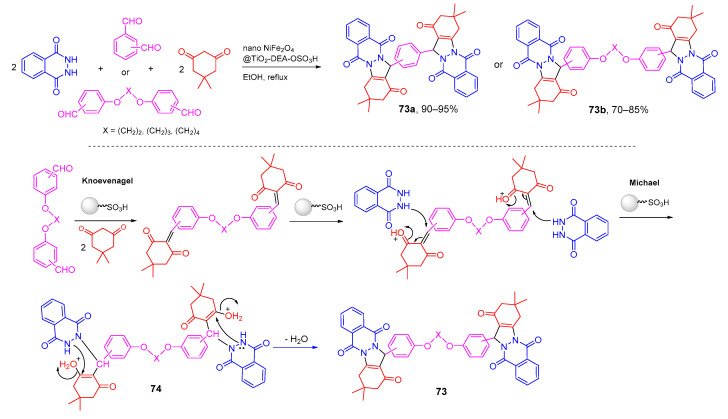
Pseudo-5CR for bis-1*H*-indazolo[1,2-*b*]phthalazine-triones.

The Hamidinasab group employed magnetic nanocatalyst NiFe_2_O_4_@TiO_2_-DEA-OSO_3_H in the synthesis of bis-1*H*-indazolo[1,2-*b*]phthalazinetriones **73a** and **73b** through a reaction of two equiv each of dimedone and phthalhydrazide and one equiv of diarylaldehydes (Scheme 29) [45]. The reaction process involves acidic nanocatalyst-promoted Knoevenagel condensation of dialdehydes and dimedones followed by Michael-type addition with phthalhydrazide to give intermediates **74**. Cyclization of **74** and tautomerization gives products **73**.

**Scheme 30:**
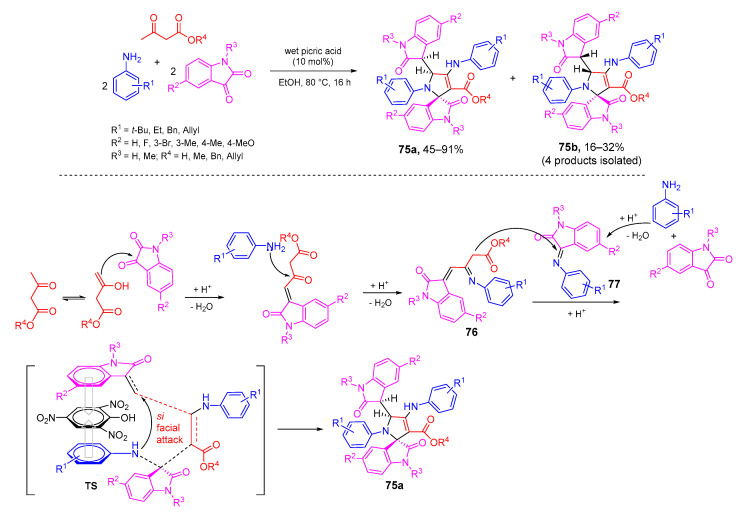
Pseudo-5CR for spiro[indole-3,2′-pyrrole] compounds.

The Mukhopadhyay group reported a method for the synthesis of highly-functionalized spiro[indole-3,2′-pyrrole] compounds **75a** using two equiv each of arylamines and isatins and one equiv of β-keto esters in the presence of wet picric acid (Scheme 30) [46]. The condensation of β-keto esters with isatins followed by condensation with arylamines give intermediates **76**. The nucleophilic addition of **76** and intermediates **77** generated from condensation of arylamines and isatins affords *syn* products **75** via *Si*-facial attack in a wet picric acid-stabilized charge transfer complex transition state.

**Scheme 31:**
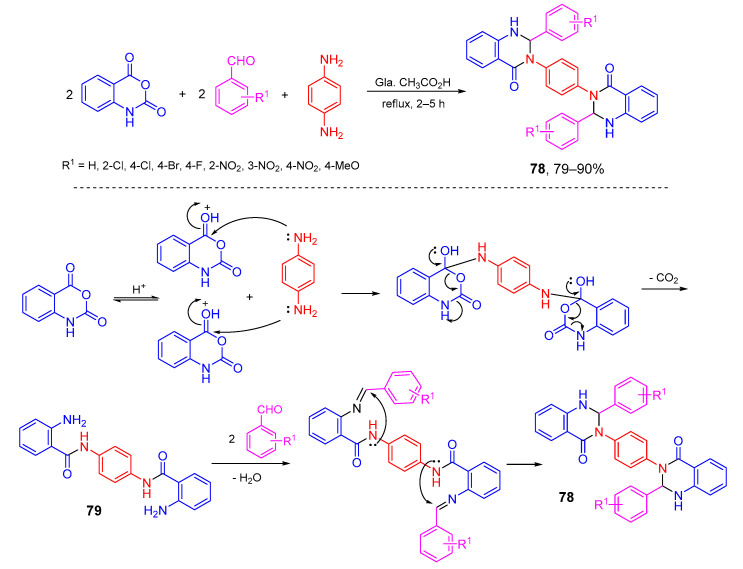
6C5CR for bis(2-phenyl-2,3-dihydroquinazolin-4(1*H*)-ones).

**Scheme 32:**
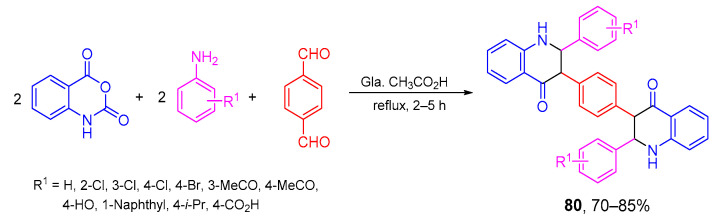
6C5CR for bis(2-phenyl-2,3-dihydroquinazolin-4(1*H*)-ones).

The Lalitha group reported a method for the synthesis of novel bis(2-phenyl-2,3-dihydroquinazolin-4(1*H*)-one) derivatives **78** using two equiv each of isatoic anhydride and aromatic aldehydes and one equiv of *p*-phenylenediamine in glacial acetic acid under reflux conditions (Scheme 31) [47]. The synthesized products have been evaluated for antioxidant property and anticancer activity. The reaction process involves a nucleophilic attack of *p*-phenylenediamine on the carbonyl group of protonated isatoic anhydride followed by decarboxylation to afford intermediate **79**. Double condensations of **79** with two aldehydes give imines for cyclization to afford products **78**. It is a 6C5CR, since *p*-phenylenediamine is a 2-centered reactant. The same group modified the reaction by using terephthaldialdehyde to replace *p*-phenylenediamine as a 2-centered reactant in the synthesis of 2,2′-(1,4-phenylene)bis(3-phenyl-2,3-dihydroquinazolin-4(1*H*)-one) derivatives **80** (Scheme 32) [47]. The Nikoofar group recently employed multi-layered nano [(Asp-Gua) IL@PEG-SiO_2_] catalyst for the synthesis of bis(2-phenyl-2,3-dihydroquinazolin-4(1*H*)-one) derivatives using *p*-phenylenediamine as a 2-centered reactant [19].

**Scheme 33:**
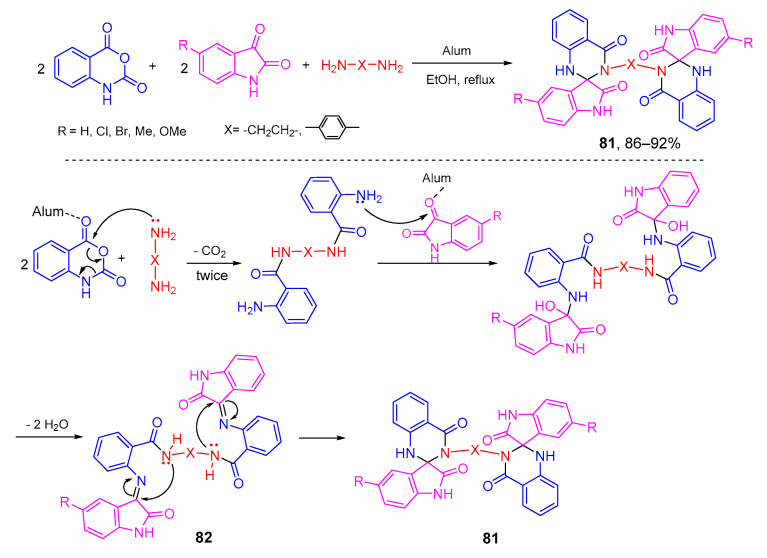
6C5CR involving isatins for bis[spiro(quinazoline-oxindole)] derivatives.

**Scheme 34:**
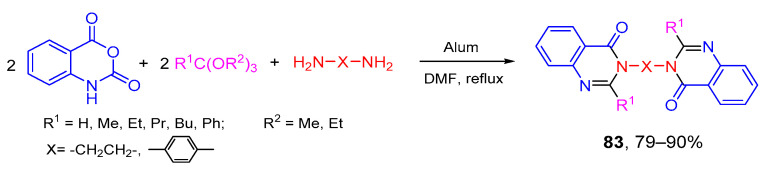
6C5CR involving orthoesters for bis(quinazolinon-4(1*H*)-one) derivatives.

The Mohammadi group developed a 6C5CR for the synthesis of novel bis[spiro(quinazoline-oxindole)] derivatives **81** using two equiv each of isatoic anhydride and isatins and one equiv of diamines under the catalysis of alum (KAl(SO_4_)_2_^●^12H_2_O) (Scheme 33) [48]. The condensation of two isatoic anhydride with ethylenediamine followed by second condensation with isatins give iminoisatins **82** after dehydration. Two intramolecular nucleophilic additions of **82** lead to the formation of products **81**. The same group applied this method for the synthesis of bis(quinazolinon-4(1*H*)-one) derivatives **83** by replacing isatins with orthoesters (Scheme 34) [49].

**Scheme 35:**
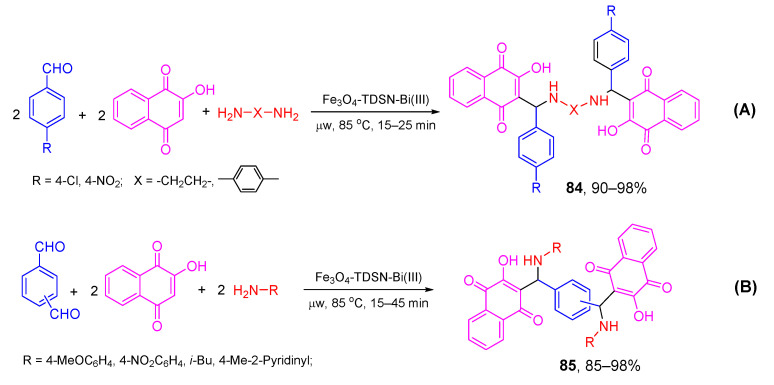
6C5CR for symmetric bis-aminonaphthoquinones. (**A**) Diamines involved reaction. (**B**) Dialdehydes involved reaction.

Mohammadpoor-Baltork and co-workers extended the 2A + B + C + D pseudo-5CR shown in Scheme 22 to a 2A + 2B + C pseudo-5CR by using diamines (Scheme 35A) or dialdehydes (Scheme 35B) as 2-centered reactants in the synthesis of symmetric bisaminonaphthoquinones **84** and **85 [32]**.

**Scheme 36:**
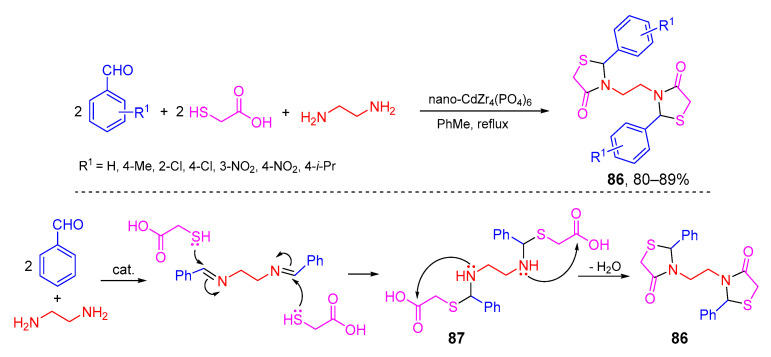
Pseudo 5-CR for the synthesis of bis-thiazolidinone derivatives.

The Safaei-Ghomi group employed a nanocrystalline nano-CdZr_4_(PO_4_)_6_ ceramic as a retrievable catalyst in the synthesis of bisthiazolidinone derivatives **86** through a reaction of two equiv each of aldehydes and thioglycolic acid with one equiv of 2-centered reactant ethylenediamine in toluene under reflux conditions (Scheme 36) [50]. The condensation of two aldehyde molecules with ethylenediamine followed by attacking of two thioglycolic acids gives **87**. The final step of double cyclization of **87** affords bisthiazolidinone products **86**.

**Scheme 37:**
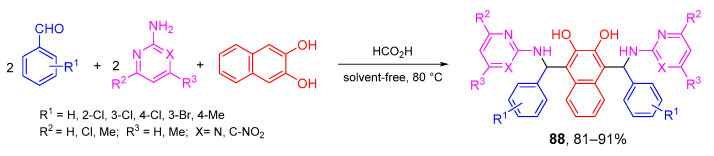
6C5CR for bis(1-aminomethyl-2-hydroxy)naphthalenes.

The Olyaei group reported a solvent-free 6C5CR for the synthesis of bisBetti bases (bis(1-aminomethyl-2-hydroxy)naphthalenes) **88** using two equiv each of aryl aldehydes and heteroaryl amines and one equiv of 2,3-dihydroxynaphthalene under the catalysis of formic acid at 80 °C (Scheme 37) [51].

**Scheme 38:**
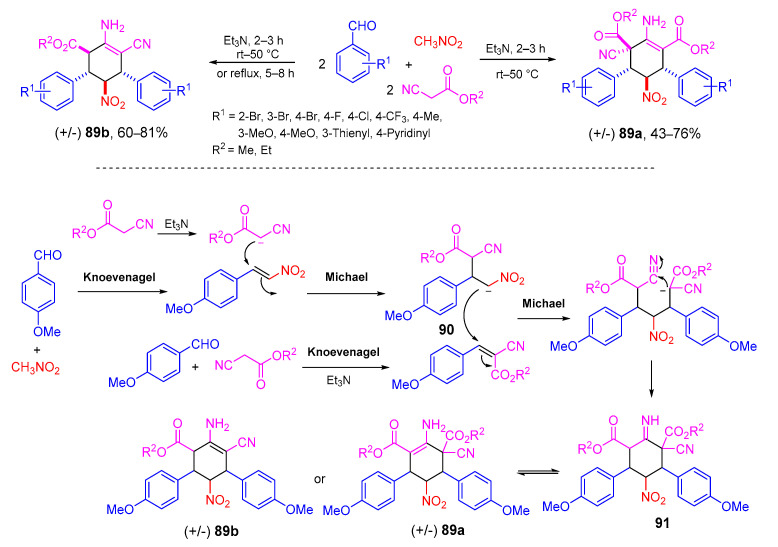
Pseudo-5CR for functionalized cyclohexene β-aminoesters.

The Wang group developed an Et_3_N-promoted reaction using two equiv each of aryl aldehydes and substituted cyanoacetates and one equiv of nitromethane to give densely functionalized cyclohexene β-aminoesters **89a** and **89b** (Scheme 38) [52]. The Knoevenagel condensation of aromatic aldehydes and nitromethane followed by the Michael addition of cyanoacetate anions affords intermediates **90.** Next, Knoevenagel condensation of aromatic aldehydes and cyanoacetate followed by the Michael addition of **90** and intramolecular nucleophilic addition affords intermediates **91** and then, products **89a** after tautomerization. Intermediates **91** could also be converted to products **89b** through decarboxylation of the *α*-carboxylic esters.

**Scheme 39:**
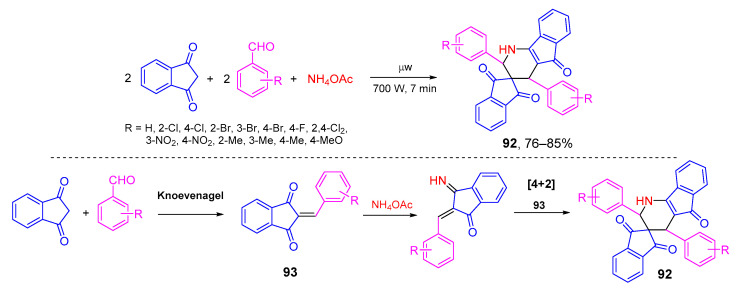
Pseudo-5CR for spiroindenotetrahydropyridine derivatives.

Prajapati and co-workers reported a microwave reaction using two equiv each of 1,3-indanediones and aromatic aldehydes and one equiv of ammonium acetate for the synthesis of novel spiroindenotetrahydropyridine derivatives **92** under catalyst- and solvent-free conditions involving cascade Knoevenagel/aza-Diels-Alder reactions (Scheme 39) [53]. The Knoevenagel condensation of indanedione and aldehydes gives dienophiles **93**. Condensation of **93** with ammonium acetate followed by aza–Diels–Alder cycloaddition of dienophiles **93** affords products **92**.

**Scheme 40:**
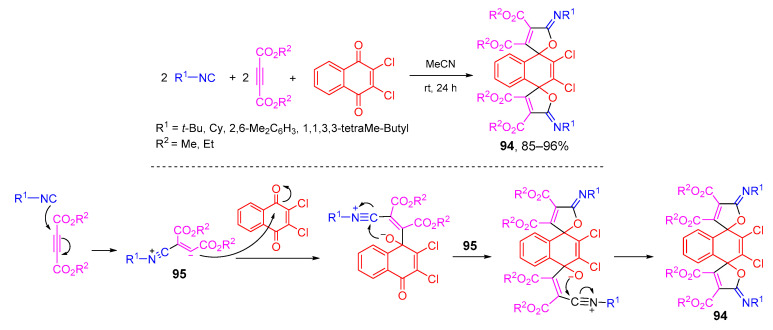
Pseudo-5CR for dispiro[furan-2,1′-naphthalene-4′,2′′-furan]tetracarboxylates.

Ghahremanzadeh and co-workers reported a reaction for diastereoselective synthesis of dispiro[furan-2,1′-naphthalene-4′,2′′-furan]tetracarboxylates **94** using two equiv each of isocyanides and dialkyl acetylenedicarboxylates and one equiv of 2,3-dichloronaphthalene-1,4-dione in acetonitrile at room temperature (Scheme 40) [54]. The isocyanides react with dialkyl acetylenedicarboxylates to form 1:1 zwitterionic intermediates **95** for nucleophilic attack at both carbonyls of 2,3-dichloronaphthalene-1,4-dione to form the species for dipolar cyclization to give products **94**.

**Scheme 41:**
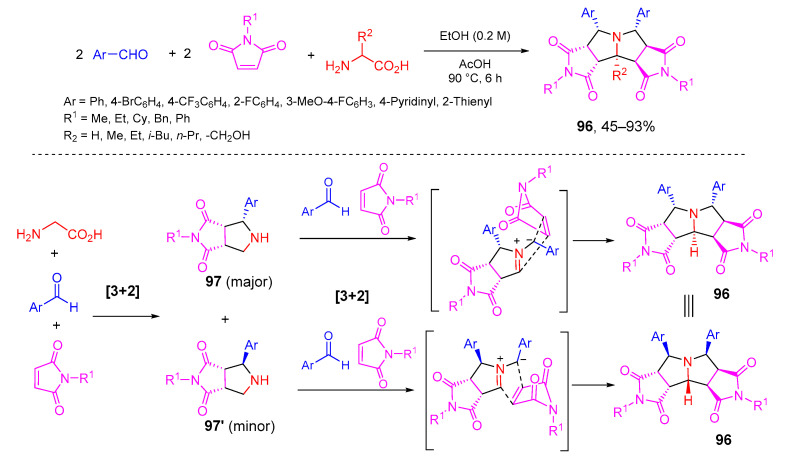
Pseudo-5CR for polycyclic pyrrolidine compounds.

**Scheme 42:**
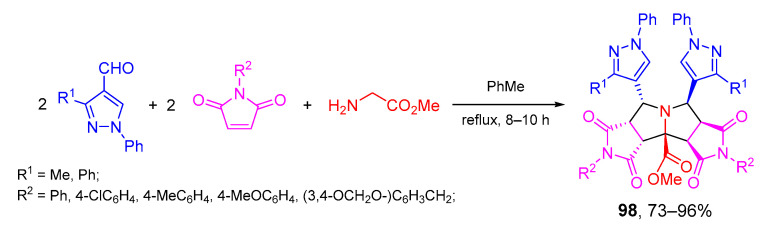
Pseudo-5CR for the synthesis of pyrazolylpyrrolizine derivatives.

The Zhang group reported the first example of a double 1,3-dipolar cycloaddition of two nonstabilized azomethine ylides for the diastereoselective synthesis of polycyclic pyrrolidines **96** using two equiv each of aromatic aldehydes and *N*-substituted maleimides and one equiv of amino acids (Scheme 41) [55]. The first decarboxylative [3+2] cycloaddition affords pyrrolidine diastereomers **97** and **97′**, which then react with aromatic aldehydes to generate a second 1,3-dipolar species for another [3+2] cycloaddition with maleimides to form pyrrolidine-containing tetracyclic compounds **96**. The same group has previously reported another double 1,3-dipolar cycloaddition using amino esters instead of amino acids [56]. The Quiroga group also reported a reaction of amino esters in the synthesis of polycyclic pyrrolidines **98** (Scheme 42) [57].

**Scheme 43:**
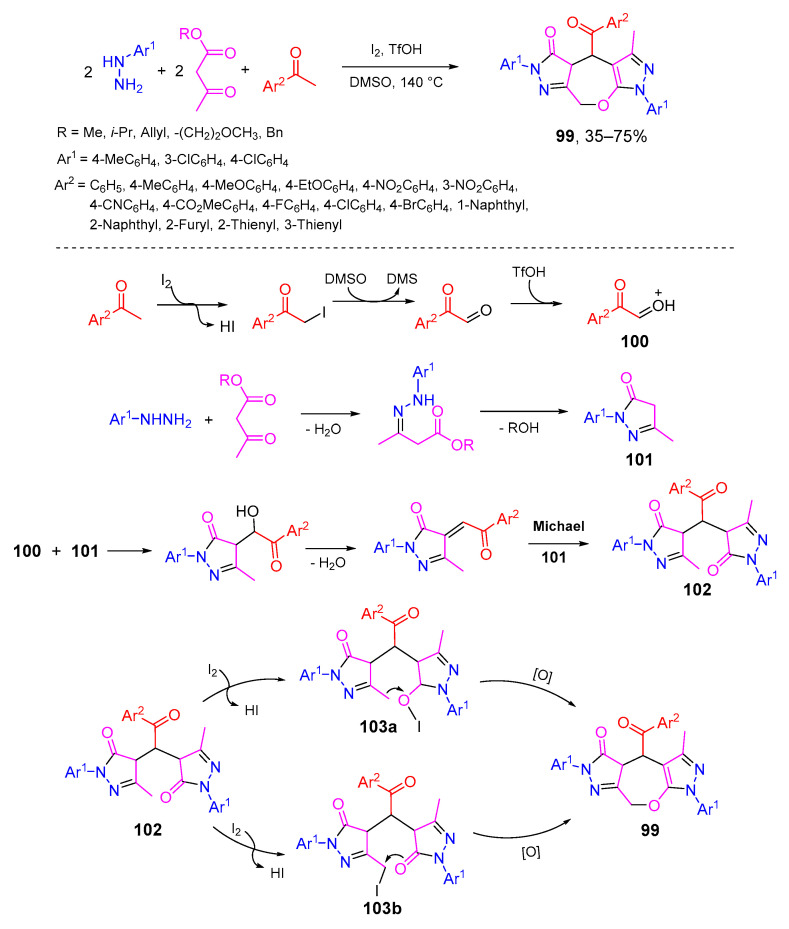
Pseudo-5CR for pyrazolone-oxepine-pyrazoles.

The Wu group developed an iodine-promoted reaction using two equiv each of phenylhydrazines and acetoacetate esters and one equiv of aryl methyl ketones for the synthesis of pyrazolone-oxepine-pyrazoles **99** (Scheme 43) [58]. Aryl methyl ketones are converted to **100** via iodination and Kornblum oxidation, while phenylhydrazines react with acetoacetate esters through dehydration condensation/aminolysis sequence to form intermediates **101**. The condensation of **100** and **101** followed by Michael addition with another molecule of **101** forms **102**. Iodination of **102** generates **103a** or **103b** followed by iodine-based oxidative coupling which affords products **99**.

Piperidine is a privileged *N*-heterocyclic ring and its derivatives possess diverse pharmacological activities such as anticancer, antimicrobial, antioxidant, anti-inflammatory, and acetylcholinesterase inhibitory activities [59]. There are several papers on the synthesis of piperidine derivatives through the reaction of aromatic aldehydes, anilines, and β-keto esters under different conditions. Catalysts used for the reactions include acetic acid [60], ethylenediammonium diformate (EDDF) [61], nanostructured PbCr_x_Fe_12−x_O_19_ [62], Fe_3_O_4_@TDSN-Bi(III) [63], anionic surfactants sodium dioctyl sulfosuccinate (SDOSS), and sodium dodecyl sulfate (SDS) [64].

**Scheme 44:**
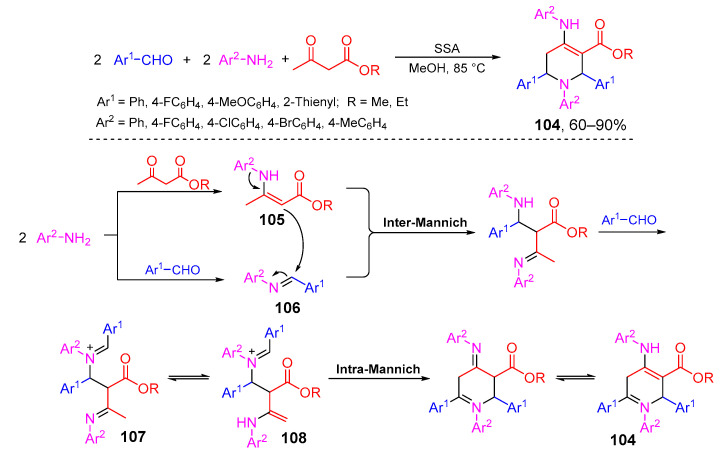
Pseudo-5CR for functionalized piperidines.

The Abbas lab reported a silica sulfuric acid (SSA)-catalyzed reaction for the synthesis of highly functionalized piperidine compounds **104** using two equiv each of aldehydes and amines and one equiv of β-ketoesters (Scheme 44) [65]. The amines reacted with β-ketoesters and aldehydes to afford β-enaminoenes **105** and imines **106,** respectively. Intermolecular Mannich reaction of **105** and **106** followed by condensation with another aldehyde affords intermediates **107**. The tautomers **108** undergo intramolecular Mannich-type reaction followed by tautomerization to generate the corresponding piperidine derivatives **104**.

**Scheme 45:**
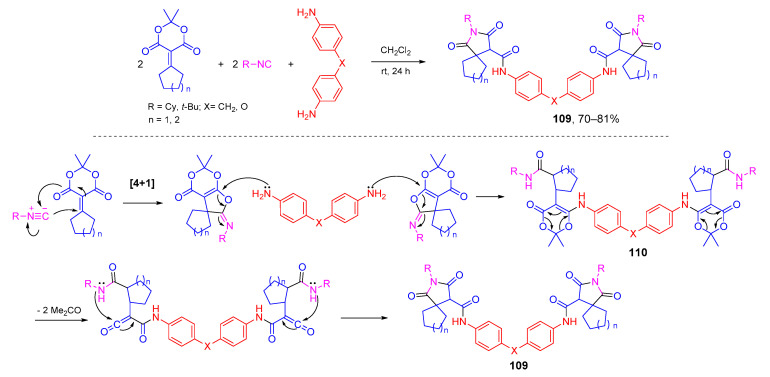
6C5CR for the synthesis of carboxamide compounds.

The Amrollahi lab reported a catalyst-free 6C5CR for the synthesis of symmetric carboxamide compounds **109** using two equiv each of alkyl isocyanides and Meldrum’s acids and one equiv of 2-centered reactant 4,4′-methylene- or 4,4′-oxydianiline (Scheme 45) [66]. Cycloaddition of isocyanides and alkylidene-substituted Meldrum’s acids followed by conjugate addition with dianilines gives intermediates **110**. The elimination of acetone via electrocyclic ring opening of Meldrum’s acid moiety of **110** followed by double cyclization delivers desired products **109**.

**Scheme 46:**
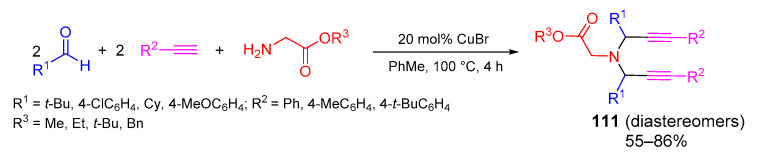
Pseudo 5-CR for tertiary propargylic amines.

Van der Eycken’s group reported an aldehyde–alkyne–amine (A^3^)-coupling reaction for the synthesis of tertiary propargylic amines **111** by integrating two molecules each of aldehydes and alkynes and one molecule of amino esters under the catalysis of CuBr in toluene at 100 °C (Scheme 46) [67].

**Scheme 47:**
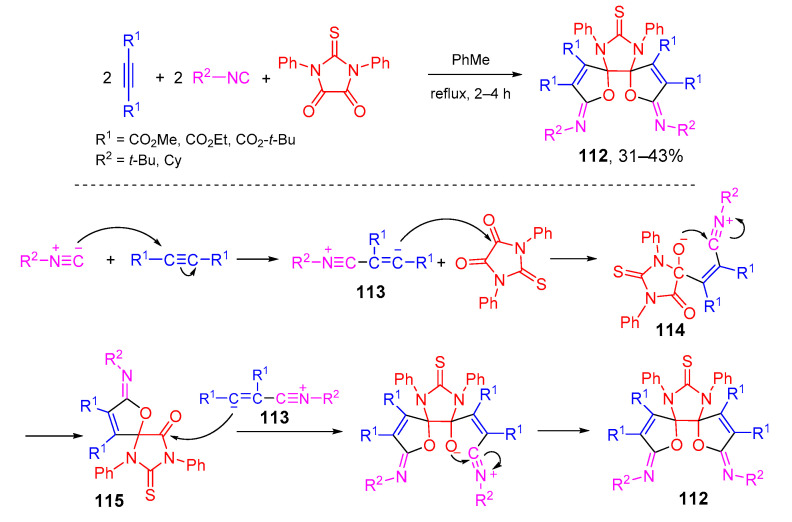
6C5CR for *γ*-dispiroiminolactones.

The Asghari lab reported an unexpected 6C5CR of two equiv each of acetylenic esters and alkyl isocyanides and one equiv of *N*,*N*’-diphenylthioparabanic acid amide to form *γ*-dispiroiminolactones **112** (Scheme 47) [68]. The reaction mechanism could involve the Michael-type reaction of isocyanides with dialkyl acetylenedicarboxylates to form reactive zwitterionic intermediates **113** which react with a carbonyl group of *N*,*N*′-diphenylthioparabanic acid amide to afford intermediates **114** for sequential cyclization to form γ-spiroiminolactones **115**. Another carbonyl group of **115** reacts with zwitterionic intermediates **113** followed by a second cyclization to give *γ*-dispiroiminolactone products **112**.

**Scheme 48:**
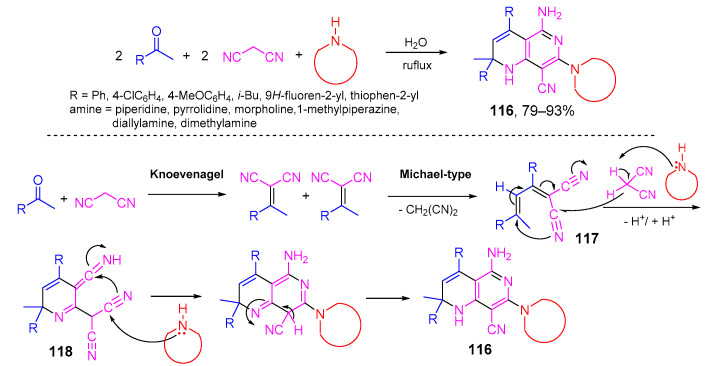
Pseudo-5CR for functionalized 1,6-naphthyridines.

Mukhopadhyay and coworkers reported a catalyst-free reaction involving Knovenagel/Michael-type addition/ring closure/cyclization/aromatization sequence for the synthesis of functionalized 1,6-naphthyridines **116** using two equiv each of methyl ketones and malononitrile and one equiv of amines (Scheme 48) [69]. Knoevenagel condensation of aromatic ketones with malononitrile followed by Michael-type reaction and subsequent elimination of malononitrile afford intermediate **117**. Malononitrile attacks **117** triggering the ring closure to form intermediates **118** which then react with an amine to produce final products **116** after aromatization. A similar process reported by the Thirumalai group using same components, but different molar ratio, gave similar products [70]. The biological evaluation results indicated that all the synthesized products possess in-vitro anti-inflammatory and antioxidant activities.

## 5. Type-IV, Pseudo-5CRs of 3A + B + C

**Scheme 49:**
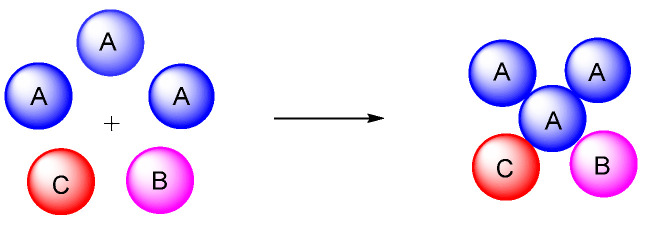
5CRs of 3A + B + C.

**Scheme 50:**
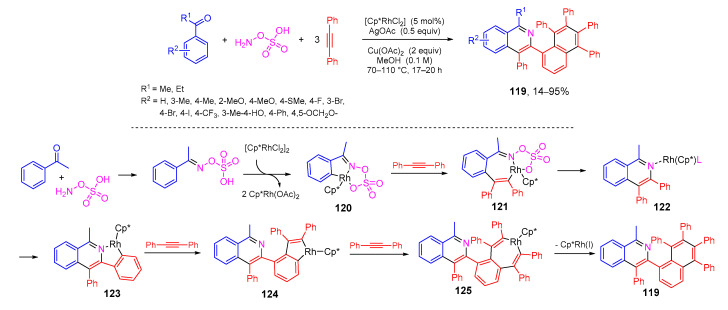
Pseudo-5CR for the synthesis of N-PAHs.

A schematic of a Type-IV pseudo-5CRs involving 3A + B + C is shown in Scheme 49. Reactions of three molecules of component A with one molecule each of B and C is very rare. The Ravikumar group reported a Rh-catalyzed reaction for the synthesis of aza-polycyclic aromatic hydrocarbons (N-PAHs) **119** using three equiv of diphenylacetylene and one equiv each of an aryl ketone and hydroxylamine-*O*-sulfonic acid (HOSA) (Scheme 50) [71]. In this reaction, the aminating reagent HOSA acts as an in situ redox-neutral directing group for the construction of N-PAHs through cascade triple C−H bond activations and multiple bond formations. The beginning of the reaction is the activation of [Cp*RhCl_2_]_2_ with AgOAc to form a rhodium catalyst which undergoes cyclometallation with *E*-(((1-phenylethylidene)amino)oxy)sulfonic acid to form **120** followed by insertion of diphenylacetylene to form **121**. Redox-neutral cyclization of **121** forms isoquinoline **122** which is converted to **123** followed by insertion of two equiv of diphenylacetylene to form **124** and then **125**. Final reductive elimination of **125** gives products **119** and N-PAHs and Cp*Rh(I) is oxidized by Cu(II) to regenerate the catalyst.

## 6. Type-V, Pseudo-5CRs of 3A + 2B

**Scheme 51:**
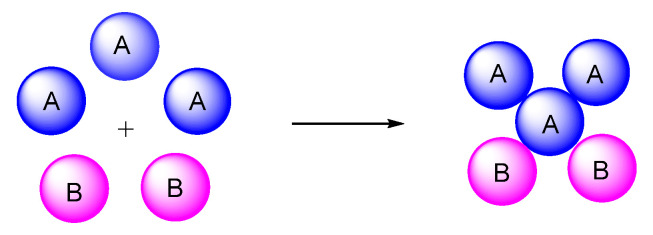
5CRs of 3A + 2B.

**Scheme 52:**
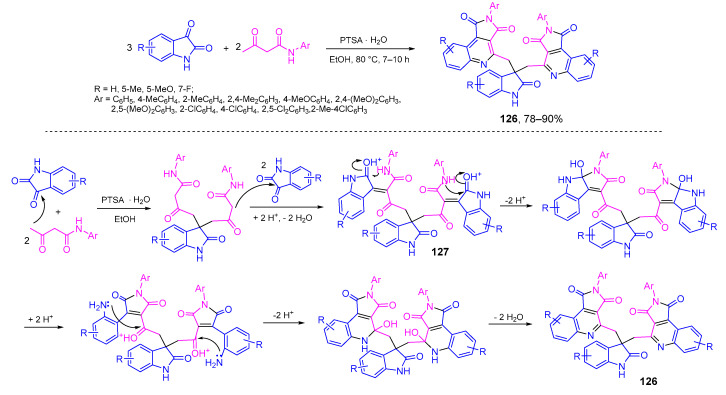
Pseudo-5CR for pyrroloquinoline derivatives.

A schematic of a Type-V pseudo-5CRs involving 3A + 2B is shown in Scheme 51. Reactions of three molecules of A with two molecules of B is a very special MCR process. Rong and co-workers developed a reaction involving three equiv of isatins and two equiv of 3-oxo-*N*-arylbutanamide for the synthesis of pyrrolo[3,4-*c*]quinoline derivatives **126** (Scheme 52) [72]. In the reaction process, isatins first react with two molecules of acetoacetanilides followed by the Knoevenagel condensation reaction with two molecules of isatins to form intermediates **127**. Intramolecular annulation of **127** and hemiaminal ring opening followed by losing two molecules of water gives products **126**.

## 7. Type-VI, Pseudo-5CRs of 4A + B

**Scheme 53:**
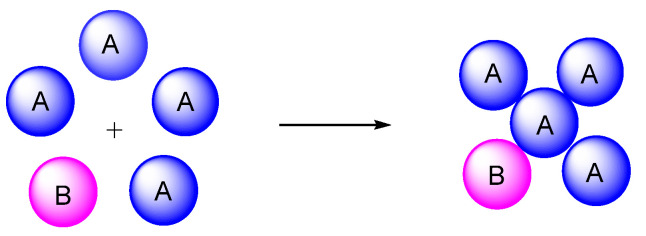
5CRs of 4A + B.

**Scheme 54:**
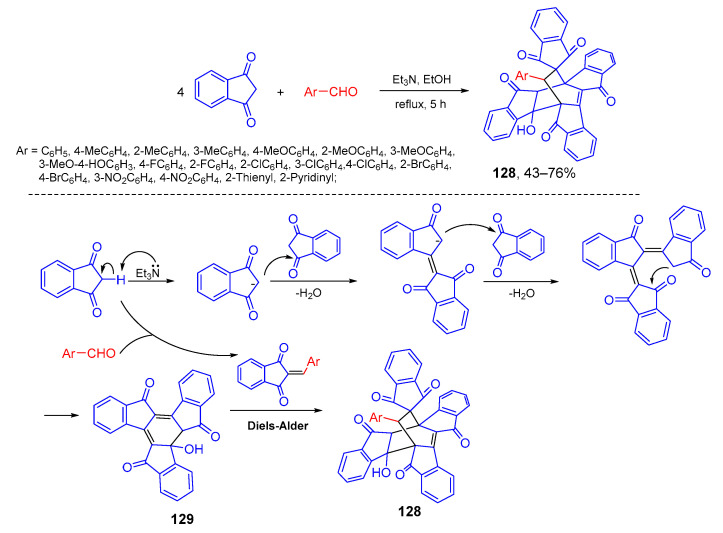
Pseudo-5CR for bicyclo[2.2.2]octane derivatives.

A schematic of a Type-VI pseudo-5CRs involving 4A + B is shown in Scheme 53. MCRs of four molecules of A with one molecule of B is a very special reaction. We only found one example from the literature. Yan, Sun, and co-workers reported a method for the synthesis of unique polycyclic bicyclo[2.2.2]octane derivatives **128**, by integrating four equiv of 1,3-indanedione and one equiv of aromatic aldehydes under Et_3_N catalysis in refluxing EtOH (Scheme 54) [73]. The reaction process involves a base-catalyzed cyclotrimerization of 1,3-indanedione to form active cyclic diene **129** followed by the *endo*-selective Diels−Alder reaction with in situ generated 2-arylidene-1,3-indanediones to give bicyclo[2.2.2]octane derivatives **128** as pure diastereomers.

## 8. Conclusions

Multicomponent reactions and associated one-pot and cascade reactions are increasing their popularity in the synthesis of complex molecules due to their inherent advantages on mass efficiency, simple operation, resource saving, and less waste disposal. Five-component reactions play a special role in MCRs. Compared to popular 3CRs and 4CRs, the number of reported 5CRs is much less and it is hard to develop new 5CRs due to competitive side reactions. However, 5CRs are more efficient in the construction of complex structures which have a large space for structural complexity and substitution diversity using commercially available starting materials such as amines/hydrazines, alcohols, azides, aldehydes/ketones, isonitriles, and carboxylic acids/esters.

Presented in this paper are six different kinds of 5CRs including five pseudo-5CRs which demonstrate the feasibility of 5CRs for the construction of complex molecules, especially polycyclic and heterocyclic molecules. The power of 5CRs could be enhanced by the following modifications: (1) performing step-wise reactions instead of addition of all components together to improve conversion and product selectivity; (2) conducting post-condensation reactions including consecutive MCRs [74], cyclization and cycloaddition reactions to access new structures with high diversity and complexity [7,8]; and (3) integrating 5CRs with other reactions, such as radical cascade reactions, photoredox-, transition metal- and organocatalysis, and electrochemical reactions. We have no doubt that 5CRs and other high-order MCRs will be unique tools in making complex molecules with potential biological and functional material applications.

## References

[1] Zhu J., Bienaymé H. (2005). Multicomponent Reactions.

[2] Zhu J., Wang Q., Wang M.-X. (2015). Multicomponent Reactions in Organic Synthesis.

[3] Herrera R.P., Marques-Lopez E. (2015). Multicomponent Reactions—Concepts and Applications for Design and Synthesis.

[4] Ameta K.L., Dandia A. (2017). Multicomponent Reactions—Synthesis of Bioactive Heterocycles.

[5] Clarke P.A., Santos S., Martin W.H.C. (2007). Combining pot, atom and step economy (PASE) in organic synthesis. Synthesis of tetrahydropyran-4-ones. Green Chem..

[6] Hayashi Y. (2016). Pot economy and one-pot synthesis. Chem. Sci..

[7] Zhang W., Yi W.-B. (2019). Pot, Atom, and Step Economy (PASE) Synthesis.

[8] Zhang W., Ballini R. (2019). One-pot Organic Reactions. Green Synthetic Processes and Procedures.

[9] Brauch S., van Berkel S.S., Westermann B. (2013). Higher-order multicomponent reactions: Beyond four reactants. Chem. Soc. Rec..

[10] Web of Science Search on “x-Component Reaction”. https://clarivate.libguides.com/webofscienceplatform/alldb.

[11] Nenajdenko V.G. (2020). Access to molecular complexity. Multicomponent reactions involving five or more components. Russ. Chem. Rev..

[12] Singh H., Sindhu J., Khurana J.M., Sharma C., Aneja K.R. (2013). A facile eco-friendly one-pot five-component synthesis of novel 1,2,3-triazole-linked pentasubstituted 1,4-dihydropyridines and their biological and photophysical studies. Aust. J. Chem..

[13] Chavan P.V., Pandit K.S., Desai U.V., Wadgaonkar P.P., Nawale L., Bhansali S., Sarkar D. (2017). Click-chemistry-based multicomponent condensation approach for design and synthesis of spirochromene-tethered 1,2,3-triazoles as potential antitubercular agents. Res. Chem. Intermed..

[14] Chavan P.V., Desai U.V., Wadgaonkar P.P., Tapase S.R., Kodam K.M., Choudhari A., Sarkar D. (2019). Click chemistry based multicomponent approach in the synthesis of spirochromenocarbazole tethered 1,2,3-triazoles as potential anticancer agents. Bioorg. Chem..

[15] Zong Y., Lang Y., Yang M., Li X., Fan X., Wu J. (2019). Synthesis of β-sulfonyl amides through a multicomponent reaction with the insertion of sulfur dioxide under visible light irradiation. Org. Lett..

[16] Govindaraju S., Tabassum S., Khan R.-R., Pasha M.A. (2017). Meglumine catalyzed one-pot green synthesis of novel 4,7-dihydro-1*H*-pyrazolo[3,4-*b*]pyridin-6-amines. Chin. Chem. Lett..

[17] Kumari S., Singh H., Khurana J.M. (2016). An efficient green approach for the synthesis of novel triazolyl spirocyclic oxindole derivatives via one-pot five component protocol using DBU as catalyst in PEG-400. Tetrahedron Lett..

[18] Faraz S.M., Rahmati A., Mirkhani V. (2017). One-pot isocyanide-based five-component reaction: Synthesis of highly functionalized *N*-cyclohexyl-2-(2,4-dioxo-2,3,4,5 tetrahydro-1*H*-benzo[*b*][1,5]diazepin-3-yl)-2-phenylacetamides. Synth. Commun..

[19] Nikoofar K., Shahriyari F. (2020). Novel bio-based core-shell organic-inorganic nanohybrid from embedding aspartic acid-guanine ionic liquid on the hydroxylated nano silica surface (nano [(Asp-Gua) IL@PEG-SiO_2_]): A versatile nanostructure for the synthesis of bis(2,3-dihydroquinazolin-4(1*H*)-one) derivatives and tricarboxamides under green media. Polyhedron.

[20] Rahmati A., Kenarkoohi T., Ahmadi-Varzaneh M. (2013). Synthesis of novel series of malonamides derivatives via a five-component reaction. Mol. Divers..

[21] Kenarkoohi T., Rahmati A. (2019). Synthesis of malonamide pseudopeptidic compounds using a pseudo five-component reaction and evaluation of their gelation properties. J. Mol. Liq..

[22] Googol F., Rahmati A. (2018). Synthesis of a new series of multifunctional dialkyl 2-(1-(alkylamino)-1,3-dioxo-3-phenylpropan-2-yl)malonates as low molecular weight supramolecular organogelators using five-component reaction. Tetrahedron.

[23] Nikbakht A., Ramezanpour S., Balalaie S., Rominger F. (2015). Efficient and stereoselective synthesis of *α*-hydrazino tetrazoles through a pseudo five-component domino reaction. Tetrahedron.

[24] Amanpour T., Bazgir A., Ardekani A.M., Ghahremanzadeh R. (2014). Pseudo five-component synthesis of 5-phenyldihydrospiro[diindenopyridine-indenoquinoxaline]dione derivatives via a one-pot condensation reaction. Monatsh. Chem..

[25] Li Y., Xue Z., Ye W., Liu J., Yao J., Wang C. (2014). One-pot multicomponent synthesis of highly functionalized piperidines from substituted β-nitrostyrenes, Meldrum’s acid, aromatic aldehydes, and ammonium acetate. ACS Comb. Sci..

[26] Attorresi C.I., Bonifazi E.L., Ramirez J.A., Gola G.F. (2018). One-step synthesis of *N*,*N*’-substituted 4-imidazolidinones by an isocyanide-based pseudo-five-multicomponent reaction. Org. Biomol. Chem..

[27] Liu H., Sun Q., Zhou Z., Liu J., Yang J., Wang C. (2013). One-pot synthesis of polysubstituted 2-piperidinones from aromatic aldehydes, nitromethane, ammonium acetate, and dialkyl malonates. Monatsh. Chem..

[28] Mobinikhaledi A., Bodaghifard M.A., Asadbegi S. (2016). A novel four- and pseudo-five-component reaction: Unexpected efficient one-pot synthesis of 4*H*-thiopyran derivatives. Mol. Divers..

[29] Vereshchagin A.N., Karpenko K.A., Elinson M.N., Minaeva A.P., Goloveshkin A.S., Hansford K.A., Egorov M.P. (2020). One-pot five-component high diastereoselective synthesis of polysubstituted 2-piperidinones from aromatic aldehydes, nitriles, dialkyl malonates and ammonium acetate. Mol. Divers..

[30] Adib M., Sheikhi E., Haghshenas P., Rajai-Daryasarei S., Bijanzadeh H.R., Zhu L.-G. (2014). A highly diastereoselective five-component synthesis of 1-(alkylimino)-5,5-dicyano-3a-aryloctahydro-3-oxacyclobuta[*cd*]pentalene-1a,2,5a,5b(2*H*,3a*H*)-tetracarboxylates. Tetrahedron Lett..

[31] Asadi B., Landarani-Isfahani A., Mohammadpoor-Baltork I., Tangestaninejad S., Moghadam M., Mirkhani V., Rudbari H.A. (2017). Microwave-assisted, regioselective one-pot synthesis of quinolines and bis -quinolines catalyzed by Bi(III) immobilized on triazine dendrimer stabilized magnetic nanoparticles. Tetrahedron Lett..

[32] Asadi B., Mohammadpoor-Baltork I., Tangestaninejad S., Moghadam M., Mirkhani V., Landarani-Isfahani A. (2016). Synthesis and characterization of Bi(III) immobilized on triazine dendrimer-stabilized magnetic nanoparticles: A reusable catalyst for the synthesis of aminonaphthoquinones and bis-aminonaphthoquinones. New J. Chem..

[33] Rahnamafar R., Moradi L., Khoobi M. (2020). Synthesis of benzo[*b*]xanthene-triones and tetrahydrochromeno[2,3-*b*]xanthene tetraones via three- or pseudo-five-component reactions using Fe_3_O_4_@SiO_2_/PEtOx as a novel, magnetically recyclable, and eco-friendly nanocatalyst. J. Heterocycl. Chem..

[34] Filian H., Kohzadian A., Mohammadi M., Ghorbani-Choghamarani A., Karami A. (2020). Pd(0)-guanidine@MCM-41: A very effective catalyst for rapid production of bis (pyrazolyl)methanes. Appl. Organometal. Chem..

[35] Noruzian F., Olyaei A., Hajinasiri R., Sadeghpour M. (2019). Guanidine hydrochloride catalyzed efficient one-pot pseudo five-component synthesis of 4,4′-(arylmethylene)bis(1*H*-pyrazol-5-ols) in water. Synth. Commun..

[36] Milani J., Maghsoodlou M.T., Hazeri N., Nassiri M. (2019). Alpha-casein: An efficient, green, novel, and eco-friendly catalyst for one-pot multi-component synthesis of bis (pyrazol-5-ols), dihydropyrano[2,3-*c*]pyrazoles and spiropyranopyrazoles in an environmentally benign manner. J. Iran. Chem. Soc..

[37] Safaei-Ghomi J., Khojastehbakht-Koopaei B., Shahbazi-Alavi H. (2014). Pseudo five-component process for the synthesis of 4,4′-(arylmethylene)bis(3-methyl-1*H*-pyrazol-5-ol) derivatives using ZnAl_2_O_4_ nanoparticles in aqueous media. RSC Adv..

[38] Sobhani S., Zarifi F., Skibsted J. (2017). Immobilized lanthanum(III) triflate on graphene oxide as a new multifunctional heterogeneous catalyst for the one-pot five-component synthesis of bis(pyrazolyl)methanes. ACS Sustain. Chem. Eng..

[39] Khaligh N.G., Mihankhah T., Gorjian H., Johan M.R. (2020). Greener and facile synthesis of 4,4′-(arylmethylene)bis(3-methyl-1-phenyl-1*H*-pyrazol-5-ol)s through a conventional heating procedure. Synth. Commun..

[40] Safaei-Ghomi J., Khojastehbakht-Koopaei B., Zahedi S. (2015). Copper chromite nanoparticles as an efficient and recyclable catalyst for facile synthesis of 4,4′-(arylmethanediyl)bis(3-methyl-1*H*-pyrazol-5-ol) derivatives. Chem. Heterocycl. Comp..

[41] Hasaninejed A., Kazerooni M.R., Zare A. (2013). Room-temperature, catalyst-free, one-pot pseudo-five-component synthesis of 4,4-(arylmethylene)bis(3-methyl-1-phenyl-1*H*-pyrazol-5-ol)s under ultrasonic irradiation. ACS Sustain. Chem. Eng..

[42] Safaei-Ghomi J., Asgari-Keirabadi M., Khojastehbakht-Koopaei B., Shahbazi-Alavi H. (2016). Multicomponent synthesis of C-tethered bispyrazol-5-ols using CeO_2_ nanoparticles as an efficient and green catalyst. Res. Chem. Intermed..

[43] Yang C., Liu P., Xu D. (2017). A green and efficient one-pot pseudo-five-component reaction for synthesis of bis(pyrazol-5-ol) derivatives via tandem cyclocondensation-Knoevenagel-Michael reaction. ChemistrySelect.

[44] Heravi M.R.P., Naghilou M. (2017). Effective preparation of 5,5′-(arylmethylene)bis(4-hydroxythiazole-2(3*H*)-one) in an aqueous fluoroalcohol solvent system under ultrasound irradiation at room temperature. Chin. Chem. Lett..

[45] Hamidinasab M., Bodaghifard M.A., Mobinikhaledi A. (2020). Synthesis of new, vital and pharmacologically important bis phthalazine-triones using an efficient magnetic nanocatalyst and their HF and NBO investigation. J. Mol. Struct..

[46] Sengupta A., Maity S., Mondal A., Ghosh P., Rudra S., Mukhopadhyay C. (2019). Pseudo five component reaction towards densely functionalized spiro[indole-3,2′-pyrrole] by picric acid, an efficient *syn*-diastereoselective catalyst: Insight into the diastereoselection on C(sp^3^)-C(sp^3^) axial conformation. Org. Biomol. Chem..

[47] Sivaguru P., Parameswaran K., Lalitha A. (2017). Antioxidant, anticancer and electrochemical redox properties of new bis(2,3-dihydroquinazolin-4(1*H*)-one) derivatives. Mol. Divers..

[48] Mohammadi A.A., Taheri S., Askari S., Ahdenov R. (2015). KAl(SO_4_)_2_^●^12H_2_O (Alum): An efficient catalyst for the synthesis of novel bis[spiro(quinazoline-oxindole)] derivatives via one-pot pseudo five-component reactions. J. Heterocycl. Chem..

[49] Mohammadi A.A., Taheri S., Askari S. (2017). One-pot pseudo five-component synthesis of some new bis(quinazolinon-4(1*H*)-one) derivatives. J. Heterocycl. Chem..

[50] Safaei-Ghomi J., Nazemzadeh S.H., Shahbazi-Alavi H. (2017). Nano-CdZr_4_(PO_4_)_6_ as a reusable and robust catalyst for the synthesis of bis-thiazolidinones by a multicomponent reaction of aldehydes, ethylenediamine and thioglycolic acid. J. Sulfur Chem..

[51] Olyaei A., Abforushha E.S., Khoeiniha R. (2017). Simple and efficient synthesis of novel bis-Betti bases via a one-pot pseudo-five-component reaction. Lett. Org. Chem..

[52] Yao J., Zhou L., Tan C., Wang C. (2015). One-pot synthesis of densely functionalized cyclic β-aminoesters containing four stereocenters, based on a Et_3_N *N*-promoted pseudo five-component reaction. Mol. Divers..

[53] Bhuyan D., Sarmah M.M., Dommaraju Y., Prajapati D. (2014). Microwave-promoted efficient synthesis of spiroindenotetrahydropyridine derivatives via a catalyst- and solvent-free pseudo one-pot five-component tandem Knoevenagel/aza-Diels–Alder reaction. Tetrahedron Lett..

[54] Sadabad H.R., Bazgir A., Eskandari M., Ghahremanzadeh R. (2014). Pseudo five-component reaction of isocyanides, dialkyl acetylenedicarboxylates, and 2,3-dichloronaphthalene-1,4-dione: A highly diastereoselective synthesis of novel dispiro[furan-2,1′-naphthalene-4′,2″-furan] derivatives. Monatsh. Chem..

[55] Zhang X., Qiu W., Evans J., Kaur M., Jasinski J.P., Zhang W. (2019). Double 1,3-dipolar cycloadditions of two nonstabilized azomethine ylides for polycyclic pyrrolidines. Org. Lett..

[56] Lu Q., Song G., Jasinski J.P., Keeley A.C., Zhang W. (2012). One-pot double [3+2] cycloaddition for diastereoselective synthesis of tetracyclic pyrrolidine compounds. Green Chem..

[57] Quiroga J., Gálvez J., Abonia R., Insuasty B., Ortíz A., Cobo J., Nogueras M. (2014). Highly efficient and diastereoselective synthesis of new pyrazolylpyrrolizine and pyrazolylpyrrolidine derivates by a three-component domino process. Molecules.

[58] Zhao P., Wu X., Geng X., Wang C., Wu Y.-D., Wu A.-X. (2018). Iodine-promoted five-component reaction using fragment assembly strategy to construct dihydrooxepines. Tetrahedron.

[59] Kaur G., Devi M., Kumari A., Devi R., Banerjee B. (2018). One-pot pseudo five component synthesis of biologically relevant 1,2,6-triaryl-4-arylamino-piperidine-3-ene-3-carboxylates: A decade update. ChemistrySelect.

[60] Lashkari M., Maghsoodlou M.T., Hazeri N., Habibi-Khorassani S.M., Sajadikhah S.S., Doostmohamadi R. (2013). Synthesis of highly functionalized piperidines via one-pot, five-component reactions in the presence of acetic acid solvent. Synth. Commun..

[61] Zarei M., Sajadikhah S.S. (2016). Green and facile synthesis of dihydropyrrol-2-ones and highly substituted piperidines using ethylenediammonium diformate (EDDF) as a reusable catalyst. Res. Chem. Intermed..

[62] Sobhani-Nasab A., Ziarati A., Rahimi-Nasrabadi M., Ganjali M.R., Badiei A. (2017). Five-component domino synthesis of tetrahydropyridines using hexagonal PbCr*_x_*Fe_12−*x*_O_19_ as efficient magnetic nanocatalyst. Res. Chem. Intermed..

[63] Asadi B., Landarani-Isfahani A., Mohammadpoor-Baltork I., Tangestaninejad S., Moghadam M., Mirkhani V., Rudbari H.A. (2017). Diastereoselective synthesis of symmetrical and unsymmetrical tetrahydropyridines catalyzed by Bi(III) immobilized on triazine dendrimer stabilized magnetic nanoparticles. ACS Comb. Sci..

[64] Parikh N., Roy S.R., Seth K., Kumar A., Chakraborti A.K. (2016). ‘On-Water’ multicomponent reaction for the diastereoselective synthesis of functionalized tetrahydropyridines and mechanistic insight. Synthesis.

[65] Basyouni W.M., El-Bayouki K.A.M., Tohamy W.M., Abbas S.Y. (2015). Silica sulfuric acid: An efficient, reusable, heterogeneous catalyst for the one-pot, five-component synthesis of highly functionalized piperidine derivatives. Synth. Commun..

[66] Mokhtari T.S., Amrollahi M.A., Sheikhhosseini E., Sheibani H. (2018). Isocyanide-based multi-component reactions: One-pot catalyst-free synthesis of carboxamides. ChemistrySelect.

[67] Sharma N., Sharma U.K., Mishra N.M., Van der Eycken E. (2014). Copper-catalyzed diversity-oriented three-and five-component synthesis of mono-and dipropargylic amines via coupling of alkynes, *α*-amino esters and aldehydes. Adv. Synth. Catal..

[68] Asghari S., Qandalee M., Sarmadi A.A. (2017). One-pot synthesis of γ-spiroiminolactones and γ-dispiroiminolactones using *N*,*N*’-disubstituted parabanic acid and thioparabanic acid derivatives. Mol. Divers..

[69] Mukhopadhyay C., Das P., Butcher R.J. (2011). An expeditious and efficient synthesis of highly functionalized [1,6]-naphthyridines under catalyst-free conditions in aqueous medium. Org. Lett..

[70] Lavanya M., Thirumalai D., Asharani I.V., Aravindan P.G. (2015). Domino synthesis of functionalized 1,6-naphthyridines and their in vitro anti-inflammatory and anti-oxidant efficacies. RSC Adv..

[71] Biswal P., Banjare S.K., Pati B.V., Mohanty S.R., Ravikumar P.C. (2021). Rhodium-catalyzed one-pot access to *N*-polycyclic aromatic hydrocarbons from aryl ketones through triple C-H bond activations. J. Org. Chem..

[72] Mao K., Dai L., Liu Y., Rong L. (2019). An efficient five–component reaction for the synthesis of 4,4′-((2-oxoindoline-3,3-diyl)bis(methylene))bis(2-aryl-1H-pyrrolo[3,4-*c*]quinoline-1,3(2*H*)-dione) derivatives. J. Heterocycl. Chem..

[73] Cao J., Shi R.-G., Sun J., Liu D., Liu R., Xia X., Wang Y., Yan C.-G. (2020). Domino reaction of aromatic aldehydes and 1,3-indanediones for construction of bicyclo[2.2.2]octanes and dibenzo[*b*,*g*]indeno[1′,2′:3,4]fluoreno[1,2-*d*]oxonines. J. Org. Chem..

[74] Zhi S., Ma X., Zhang W. (2019). Consecutive multicomponent reactions for the synthesis of complex molecules. Org. Biomol. Chem..

